# Current Collector Engineering for New Efficient Bioresorbable Sodium‐Ion Batteries

**DOI:** 10.1002/advs.76376

**Published:** 2026-06-28

**Authors:** Bincy Lathakumary Vijayan, Eleonora Vandini, Vedi Kuyil Azhagan Muniraj, Hussien Hammoud, Oussama Jamai, Eve Djenizian, Marc Ramuz, Daniela Giuliani, Manuela Zavatti, Yann Tison, Hervé Martinez, Lucas Teolis, Esma Ismailova, David Moreau, Sébastien Maria, Thierry Djenizian

**Affiliations:** ^1^ Mines Saint‐Etienne Department of Flexible Electronics Center of Microelectronics in Provence Gardanne France; ^2^ Department of Biomedical, Metabolic and Neural Sciences University of Modena and Reggio Emilia Modena Italy; ^3^ CNRS IPREM Université De Pau et Des Pays de L'adour Pau France; ^4^ Centrale Casablanca Research Center for Complex Systems and Interactions Bouskoura Ville Verte Marocco; ^5^ Université Paris‐Saclay Centralesupélec Gif‐sur‐Yvette France; ^6^ Mines Saint‐Etienne, Institut Des Neurosciences de La Timone Department of Bioelectronics Center of Microelectronics in Provence Gardanne France; ^7^ CNRS ICR UMR 7273 Aix Marseille Univ Marseille France; ^8^ Center of Physical‐Chemical Methods of Research and Analysis Al‐Farabi Kazakh National University Almaty Kazakhstan

**Keywords:** implantable batteries, Mo‐based current collector, Na‐ion technology, temporary medical implants, thin‐film metal deposition

## Abstract

Developing bioresorbable energy sources that eliminate the need for secondary surgery for device removal remains a major challenge in resorbtronics. In this work, we present an alternative fabrication strategy for new bioresorbable quasi‐solid Na‐ion batteries using Mo or Mg thin films as current collectors. The assembled batteries were characterized using scanning electron microscopy, spectroscopic techniques including X‐ray photoelectron spectroscopy, physicochemical, and electrochemical characterization techniques. Results demonstrate that the choice of current collector strongly influences electrochemical performance. Mo‐based batteries delivered a discharge capacity of 6.8 mAh cm^−^^2^ at a C/2 rate, approximately twice that of Mg‐based counterparts whose performance is limited by oxidation reactions. Moreover, Mo‐based batteries exhibited stable cycling with 86% capacity retention after 100 cycles at 2C. In vitro cytotoxicity assays showed cell viability above the 70% threshold indicating that the tested materials are non‐cytotoxic according to ISO 10993 guidelines. Implantation studies confirmed safe degradation of Mo‐based batteries supported by in vivo monitoring of animal health behavior and *ex vivo* organ analyses, including organ weight, ALT measurements, and histological evaluation of skin at the implantation site after three months. Additionally, the operational lifetime of implanted batteries can be tuned from days to months by controlling encapsulation layer thickness.

## Graphical abstract

A bioresorbable Na‐ion battery is proposed with Mo or Mg thin‐film current collectors for temporary implants. Mo‐based batteries exhibit superior electrochemical performance and stable cycling compared with Mg counterparts. XPS was used to investigate the Mo/Mg surface oxidation. Safe degradation of the battery was confirmed through in vitro and in vivo studies, while battery lifetime can be tuned via encapsulation thickness.

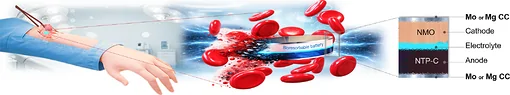

## 1 Introduction

Nowadays, healthcare has gone through remarkable advancements with the modification of biomedical implants, including pacemakers, cochlear implants, and visual prostheses, which have a significant impact on the quality of life of patients worldwide [1, 2, 3, 4]. Among these advancements, temporary medical devices (TMDs) have arisen as a promising solution for therapeutic support over a limited timeframe, which are actively being developed to assist in various medical treatments, such as electrical stimulators for nerve regeneration [5], stroke recovery [6], and bone fracture healing [7, 8]. Nevertheless, a major limitation of conventional TMDs is the necessity of a secondary surgery to remove them once they have completed their function, which increases the risk of infection followed by additional discomfort to the patients [2, 9]. To overcome these challenges, there is an urge to design the next generation implantable system, which contains bioresorbable electronics, including electrodes, sensors, energy storage devices, and integrated circuits [10, 11, 12]. These implantable devices for resorbtronics must dissolve safely into biological fluids and be resorbed by the body after their intended use, without leading to the post‐treatment complication [3, 6, 10, 11, 12, 13, 14, 15]. In the scenario, several bioresorbable electronics are reported, in which the bioresorbable materials are integrated with biological fluids, allowing direct contact with targeted molecules to enhance sensing and monitoring [16]. Moreover, bioresorbable electronics have enabled several medical treatments through the continuous monitoring of post‐surgical infection detection, trauma recovery, chemotherapeutic drug effects, pharmacokinetics analysis, and biomarker tracking [17].

Bioresorbable energy storage solutions are one of the essential components in bioresorbable electronic systems, remaining as a critical research area for implantable applications [17, 18, 19, 20, 21]. The reported bioresorbable energy storage solutions include biodegradable electrochemical capacitors with Mg and transient polymer electrolytes, silicon‐based transient capacitors, bioresorbable polymer‐based supercapacitors, and transient and primary batteries [6, 22, 23, 24, 25]. Thus, current efforts are focused on bioresorbable materials and on the structural design of bioresorbable energy storage, working for short time periods and finally being disintegrated and resorbed by the body [13]. The design and engineering of bioresorbable rechargeable batteries with the same characteristics have not been explicitly reported so far.

Currently available bioresorbable batteries are mainly primary biodegradable batteries, with a sacrificial anode metal like Mg and cathode metals such as Fe, Mo, and W which will be working with a biodegradable electrolyte. The sacrificial Mg anode, which is a major body mineral, has a minimum daily intake of 100 mg day^−1^ [26], whereas, the average dietary intake of molybdenum by adult men and women is 109 and 76 µg day^−1^, respectively [27]. There are several biodegradable batteries reporting Mo as the cathode electrode and current collector, even though the complete disintegration was achieved only after the application of an elevation in temperature to 85°C to accelerate the dissolution, which is completely far from the animal body concept [28]. Very recently, an alternative fabrication process has been implemented to fabricate a bioresorbable Na‐ion battery showing no sign of toxicity after being disintegrated under in vivo conditions [10]. In the study, the operating days of implanted batteries are demonstrated and the lifetime of this novel energy storage system after being implanted can be finely controlled by tuning the thickness of the encapsulation layer, paving the way to the design of “on‐demand” bio‐eliminable batteries that may operate for days or several weeks. However, the high reactivity of Mg thin film used as current collector is responsible for the loss of battery performance. Building on this approach, the present work investigates the influence of the current collector material by systematically comparing Mg and Mo thin‐film collectors in bioresorbable Na‐ion batteries.

In this study, a novel Na‐ion battery using Na‐CMC electrolyte in a quasi‐solid form was designed to overcome the limitations of existing bioresorbable power sources. Particularly, the critical challenges associated with developing bioresorbable rechargeable batteries are addressed by investigating the key role of thin‐layer current collectors. An alternative fabrication strategy is implemented to prevent the oxidation of bioresorbable current collectors, ensuring the stability and efficiency of the battery. The superior properties of Mo layer over the Mg counterpart are demonstrated, highlighting the detrimental impact of the severe oxidation of the Mg current collector. By employing fully biocompatible materials, the proposed bioresorbable battery can be safely eliminated by biological fluids. The biocompatibility of the batteries was assessed by in vitro cytotoxicity tests. Implantation studies confirmed the safe degradation of the Mo‐based battery, as verified through in vivo and ex vivo analysis. The present study demonstrates that the operational lifetime of implanted batteries can be tuned from days to months by controlling the thickness of an encapsulation layer. The results demonstrate a significant advancement in bioresorbable energy storage, paving the way for future developments in transient medical devices.

## 2 Results and Discussion

### 2.1 Morphological and Chemical Analysis of Bioresorbable Batteries

The morphological and chemical analysis of the Na‐ion batteries was performed by SEM, associated with EDX and XPS. Figure 1a,b shows the SEM images of Mo and Mg layers deposited on NMO and NTP‐C electrode surfaces, respectively. The electrode surfaces of NMO and NTP‐C produced by the hydraulic press exhibit sufficient smoothness to serve as an ideal substrate for the uniform deposition of thin metallic layers. These uniform metal coatings, serving as the current collectors, are expected to provide optimal electrical conductivity, ensuring stable battery operation. The quantitative results of constituent elements obtained by EDX from the surface of both Mo and Mg on NMO pellets have been plotted in Figure 1c,d. Clearly, the amount of O element is higher for the Mg‐coated NMO. Although the depth analyses by EDX is larger than the film thickness, this quantitative analysis strongly suggests that Mg is strongly oxidized. A photograph of the Mg and Mo‐coated NMO electrodes is shown in Figure 1e.

**FIGURE 1:**
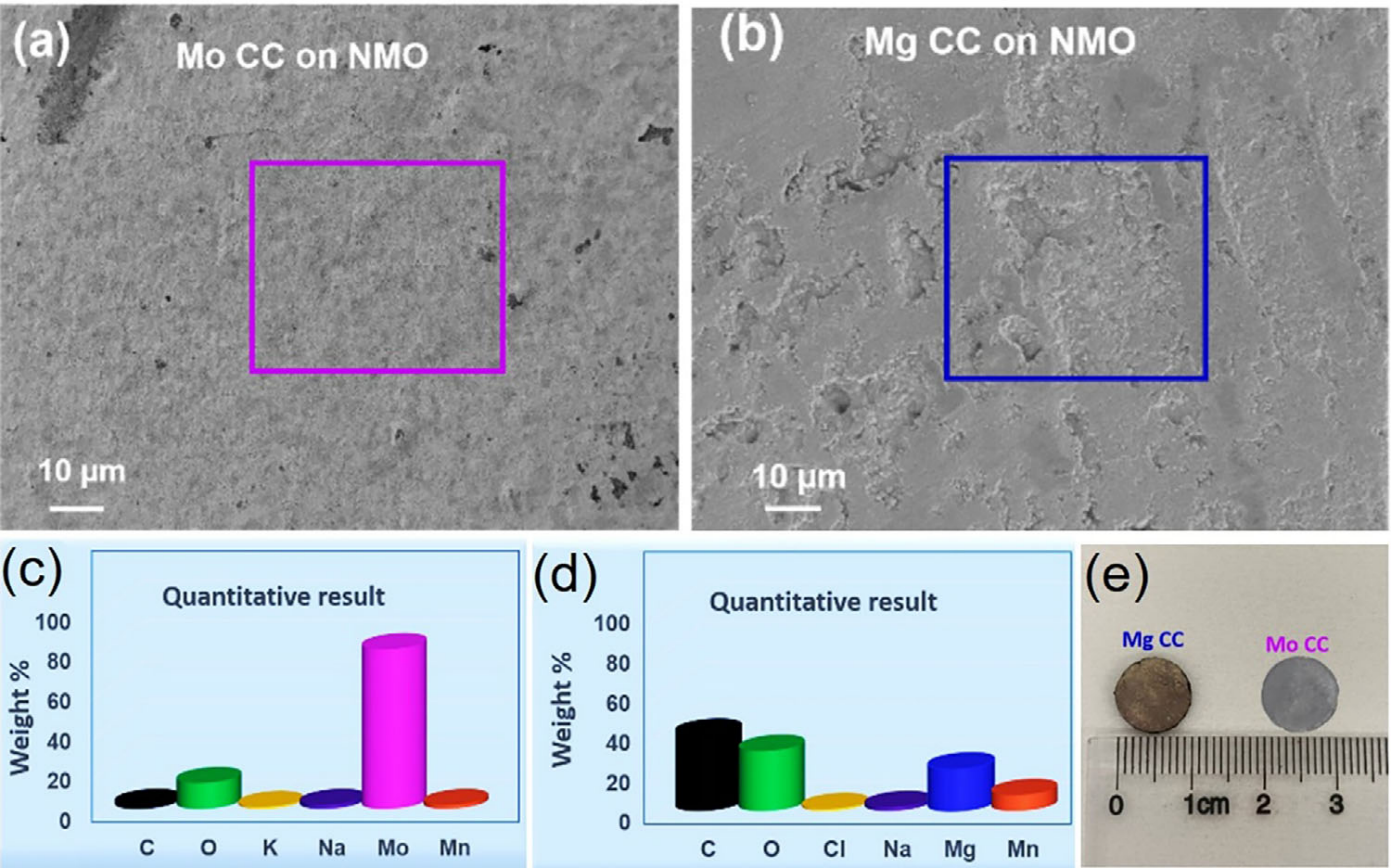
SEM images of (a) Mo layer on NMO, (b) Mg layer on NMO. (c, d) weight% of elements determined by EDX. (e) Photograph of Mo and Mg deposited on NMO electrodes.

To examine the electrode/electrolyte interfaces, the assembled battery was frozen, cut, and polished. Figure 2a shows the schematic representation of a full cell to understand the complete structure before cutting and polishing. The cross‐sectional SEM image of the battery core (Figure 2b) displays a well‐integrated ∼20 µm thick quasi‐solid‐state electrolyte (QSE) in contact with both the NMO and NTP‐C electrode surfaces. This compact interface is crucial for facilitating efficient ionic transport and ensuring stable battery performance. Additionally, EDX chemical mapping (Figure 2c) confirmed the distribution of key elements throughout the battery core, including Mn from NMO, Na from the electrolyte, Ti and P from NTP‐C, along with C and O.

**FIGURE 2:**
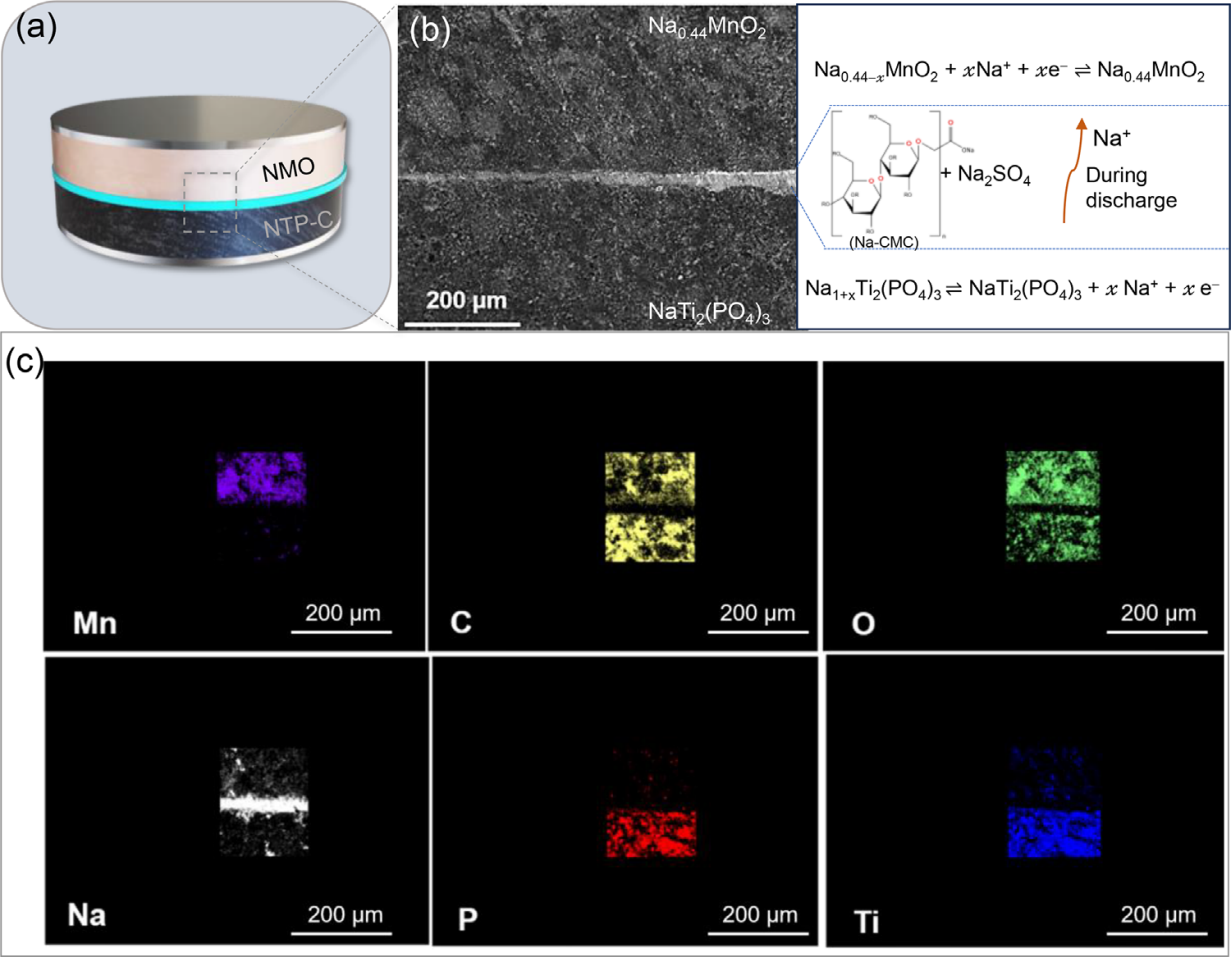
(a) Schematic representation of a bioresorbable battery, (b) cross‐sectional SEM view of the electrode/electrolyte interfaces, showing an electrolyte layer of approximately 20 µm thickness (scale bar: 200 µm). (c) Chemical mapping by EDX showing the presence of predominant elements in the different parts of the battery cross‐section.

XPS depth profiles were undertaken to identify the chemical environments of Mg and Mo in the current collectors before electrochemical cycling and the corresponding composition profiles are presented in Figure 3.

**FIGURE 3:**
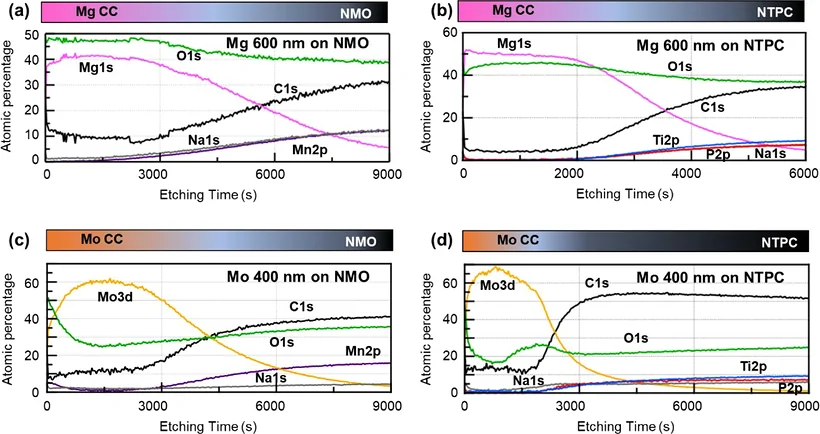
XPS concentration profiles of (a) Mg CC on NMO, (b) Mg CC on NTPC, (c) Mo CC on NMO, and (d) Mo CC on NTPC. The variation of Mg fraction is represented pink, Mo fraction in orange, Na fraction in gray, Mn fraction in purple, Ti fraction in blue, P fraction in red, O fraction in green and C fraction in black. Above each profile is a visual guide (pink represents the Mg CC, orange the Mo CC, black is the electrode and gray areas correspond to the transition region).

For the Mg current collector on both NMO (Figure 3a) and NTP‐C (Figure 3b), the atomic percentage of Mg is relatively constant ‐ca. 40% for Mg CC on NMO and ca. 50% for Mg CC on NTP‐C‐ for the first etching cycles. Then, it decreases while the percentages of Na, Mn, C for NMO and Na, Ti, P, C for NTP‐C increase. For both Mg current collectors, the oxygen content remains high ‐ca. 45% for Mg CC on NMO and ca. 40% for Mg CC on NTP‐C. Regarding Mo current collectors, the profiles mostly follow a trend similar to that of Mg. Both Mo concentrations initially increase before stabilizing at ca. 60% for Mo CC on NMO (Figure 3c) and at ca. 65% for Mo CC on NTP‐C. Then, a decrease in the Mo contents is observed, together with an increase in the fractions of Na, Mn, C for Mo CC on NMO and Na, Ti, P, C for Mo CC on NTP‐C. However, the main difference is the behavior of the oxygen ratios, which strongly decrease during the first cycles and then remain at a low value (between 20 and 30%) when the Mo content dominates before increasing when the analyzed surface corresponds to the electrodes.

Such a discrepancy in the oxygen contents when comparing Mg CC with Mo CC suggests that the Mg CC is fully oxidized, while the Mo CC is, at least partially, in the metallic state. To confirm these hypotheses, the Mg 1s core peaks, Mg 2p core peaks, Mg KLL Auger peaks, and Mo 3d core peaks on both NMO and NTP‐C have been extracted at the beginning and after 2010s of etching, as an example of etching for which the bulk of the current collector is investigated. They are reported in Figure 4.

**FIGURE 4:**
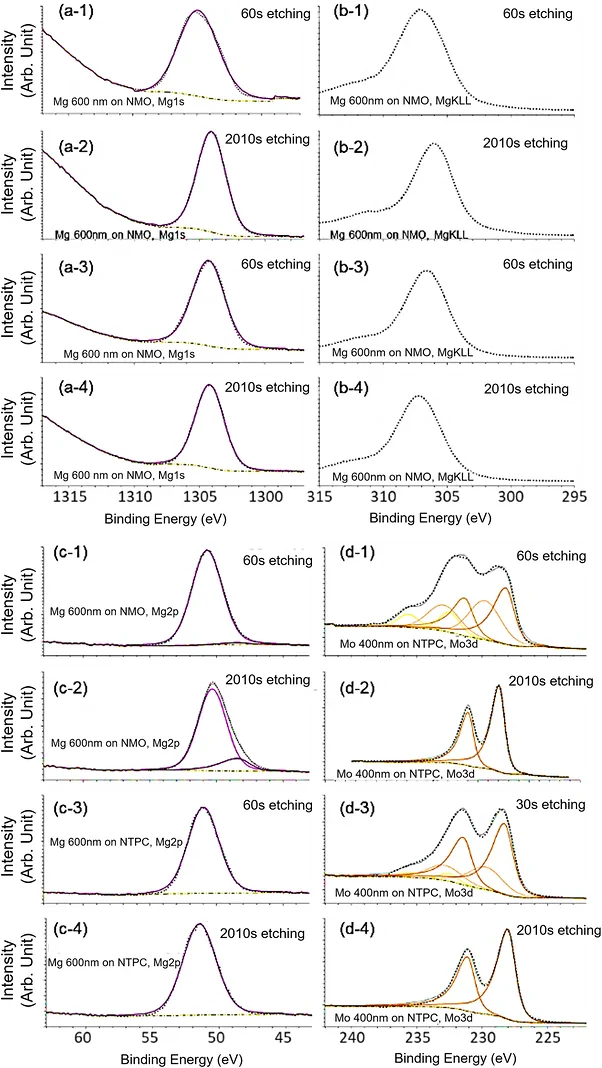
XPS spectra of Mg and Mo current collectors on NMO and NTP‐C at the initial stage of the depth profiles (30 or 60 s) and in the bulk of the current collectors (2010 s), (a‐1) Mg 1s peak on NMO after 60 s etching, (a‐2) Mg 1s peak on NMO after 2010 s etching, (a‐3) Mg 1s peak on NTPC after 60 s etching, (a‐4) Mg 1s peak on NTPC after 2010 s etching; (b‐1) Mg KLL peak on NMO after 60 s etching, (b‐2) Mg KLL peak on NMO after 2010 s etching, (b‐3) Mg KLL peak on NTPC after 60 s etching, (b‐4) Mg KLL peak on NTPC after 2010 s etching; (c‐1) Mg 2p peak on NMO after 60 s etching, (c‐2) Mg 2p peak on NMO after 2010 s etching, (c‐3) Mg 2p peak on NTPC after 60 s etching, (c‐4) Mg 2p peak on NTPC after 2010 s etching; (d‐1) Mo 3d peak on NMO after 60 s etching, (d‐2) Mo 3d peak on NMO after 2010 s etching, (d‐3) Mo 3d peak on NTPC after 30 s etching, (d‐4) Mo 3d peak on NTPC after 2010 s etching. Experimental data is shown as a dotted line, Mg 1s and Mg 2p as pink lines, Mn 3p as purple lines, the Mo 3d is decomposed into 3 doublets: Mo^6+^(232.0–235.2 eV) in yellow, Mo^4+^ (229.4–232.6 eV) in light orange and Mo^0^ (228.2–231.4 eV) in dark orange; the Shirley‐type background is presented as a brown dash‐dot line.

For both types of electrodes and both at the surface (30 or 60s etching time) or in the bulk of the Mg CC (2010s etching time), the Mg 1s spectra display single peaks centered between 1304.0 and 1305.0 eV, the X‐ray induced Mg KLL Auger peak is characterized by a single large contribution with a maximum around 306–307 eV (or 1179–1180 eV if expressed in kinetic energy) on panels a‐2, b‐2, c‐2 and d‐2 of Figure 4 and finally the Mg 2p peaks are found at 50.7 ± 0.4 eV on panels a‐3, b‐3, c‐3 and d‐3 of Figure 4. These values are indicative of Mg^2+^ ion [29], probably in the form of Mg(OH)_2_ or MgO. It is to be noted that a small Mn 3s peak is observed at ca. 48 eV in the Mg 2p regions. No evidence of metallic Mg is observed, which would be evidenced by a Mg KLL Auger peak at 302 eV in the binding energy scale (1185 eV in the kinetic energy scale) [29]

Considering Mo CC, the behavior indicated by the Mo 3d core peak is rather different: for both NMO and NTP‐C substrates, at the initial stage (30s for Mo on NTPC or 60 s for Mo on NMO, we chose to show the Mo 3d peak of the Mo on NTPC after 30 s because this sample is, for some unidentified reason, etched faster than the other samples), Figure 4 panels c‐1 and c‐3, of etching time, one can observe three doublets at 232.0–235.2, 229.4–232.6, and 228.2–231.4 eV which, in agreement with data in the literature [30], can be assigned to Mo^6+^ ions, Mo^4+^ ions and Mo in the metallic state, respectively. After 2010 s of etching (Figure 4 panels c‐2 and c‐4), corresponding to the bulk of the Mo CC, the Mo3d spectra only show a doublet at 228.0–230.0 eV with asymmetric line shapes, which is typical of metallic molybdenum [30]. Therefore, the molybdenum films appear to be in the metallic state with some surface oxidation. For both Mg and Mo films, it is to be noted that a small Mn 3s peak is observed at ca. 48 eV in the Mg 2p regions, indicating that a minute amount (less than 5 atomic percent) of manganese is found at the surface of the sample. This contribution is not observed after a few cycles of etching is most probably due to some contamination coming from Na_0.44_MnO_2_ below during the elaboration of the Mg or Mo film. As a conclusion for this chemical analysis, both the depth profiles and the Mg and Mo XPS peaks show that the Mg current collector is fully oxidized, while the Mo current collector is metallic.

### 2.2 Electrochemical Characterizations

The ionic conductivity of the quasi‐solid‐state electrolyte (QSE) made of Na_2_SO_4_/Na‐CMC (with salt‐to‐polymer ratio of 4:1) was measured at room temperature (25°C) using electrochemical impedance spectroscopy (EIS). The QSE exhibited an ionic conductivity of 1.41 × 10^−^^4^ S cm^−^^1^. To clarify the hydration behavior of the electrolyte, as shown in Figure S1, thermogravimetric analysis (TGA) was first performed on the gel electrolyte (before drying) over an extended temperature range up to 200°C. This analysis revealed that the primary mass loss associated with free (physically absorbed) water occurs up to ∼104°C, identifying this temperature range as the critical dehydration region governing ionic conductivity. Based on this observation, TGA measurements for the optimally dried quasi‐solid‐state electrolyte were conducted up to 120°C, specifically targeting the temperature range relevant to the removal of residual free water, which plays the dominant role in ionic transport in gel polymer electrolytes. The QSSE exhibits a reduced mass loss of approximately ∼20 wt.% up to ∼105°C, indicating moist remains within the Na‐CMC polymer–salt matrix upon controlled drying.

In addition, differential scanning calorimetry (DSC) analysis of the QSSE provides complementary thermal information. The DSC curve exhibits a broad endothermic peak centered around ∼130°C–135°C, which is attributed to the removal of chemically coordinated water (dehydration) associated with strong interactions between Na^+^ ions and hydrophilic functional groups of the Na‐CMC polymer matrix. These chemically coordinated functional groups (–COO^−^ and –OH/–OR) promote ion–dipole interactions and hydrogen bonding with water molecules, resulting in stronger water retention within the polymer–salt matrix and causing their removal at higher temperatures compared to free water. The combined TGA DSC results therefore confirm the presence of both free and coordinated water within the electrolyte system. The residual free water plays a significant role in facilitating ionic conductivity, while coordinated water stabilizes hydrated ionic domains within the polymer network. These hydrated polymer domains facilitate Na^+^ transport, which is consistent with the measured ionic conductivity value for the optimally dried quasi‐solid‐state electrolyte. Thus, the optimally dried condition, was selected for all subsequent experiments, as it provides a suitable compromise between ionic conductivity and structural stability of the electrolyte layer during device fabrication and operation. In Figure 5, the electrochemical performance comparison of batteries consisting of Mo CC and Mg CC was evaluated through galvanostatic charge‐discharge (GCD) measurements, rate capability tests, cycling stability analysis, and electrochemical impedance spectroscopy (EIS). As shown in Figure 5a, the initial GCD curves at a C/10 rate potential range of 0 to 1.5 V reveal that Mo CC based batteries exhibit a significantly higher discharge capacity (8.8 mAh cm^−^^2^) compared to the batteries using Mg CC (4.6 mAh cm^−^^2^), highlighting its superior charge storage capability. Even though both the bioresorbable batteries are made of same components and mass loading, the discharge capacity of the battery with Mo CC is nearly twice of the one based on Mg CC. Such effect cannot be explained by the electrical properties of the two metals; indeed, the electrical conductivities of Mo is 2 × 10^7^ S m^−1^ and Mg is 2.3 × 10^7^ S m^−1^ are quite similar in their metallic states. The capacity difference is certainly caused by the strong reactivity of Mg and oxidation effects. Actually, XPS and EDX results show the complete oxidation of Mg to probably Mg(OH)_2_, which possesses low electrical conductivity [31], hindering the electron transfer.

**FIGURE 5:**
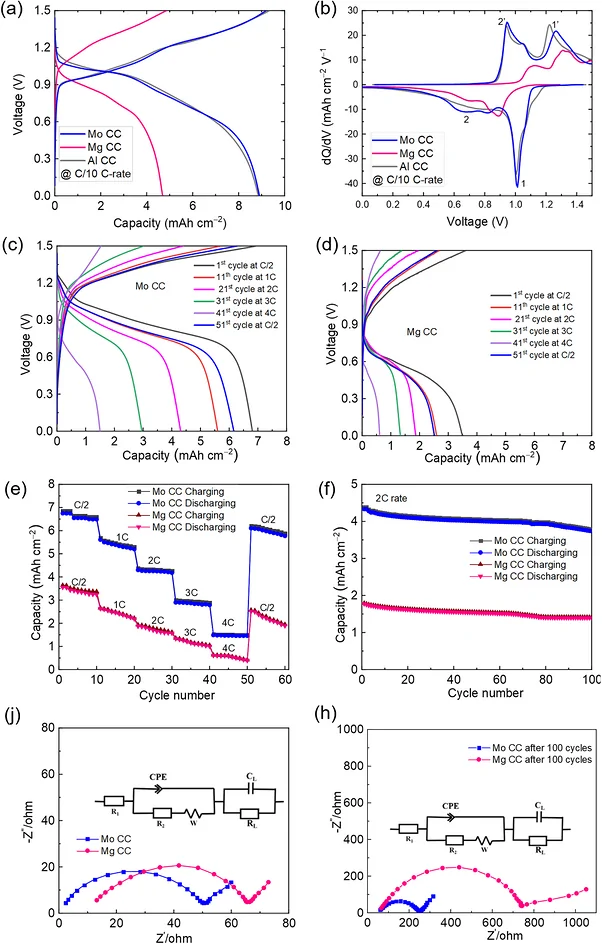
Electrochemical performances of bioresorbable Mo CC‐ and Mg CC based batteries compared with a conventional Al current collector (taken as reference). (a) First Galvanostatic charge‐discharge curves at C/10 rate. (b) Derivative plots of the charge‐discharge curves. (a) First Galvanostatic charge‐discharge curves at C/10 rate. (b) Derivative plots of the charge‐discharge curves. Galvanostatic charge‐discharge curves at various charge kinetics during 51 cycles for (c) Mo CC based battery and (d) Mg CC based battery. (e) Comparison of electrochemical performance at different C‐rates for 60 cycles. (f) Electrochemical performance at 2C rate for 100 cycles. Comparison of Nyquist plots from EIS studies at a frequency range of 10 kHz to 1 Hz and the corresponding equivalent circuits (g) before cycling and h) after 100 cycles.

As shown in Figure 5b, the derivative plots of the charge‐discharge dQ/dV profiles obtained with Mo CC exhibit identical redox peak positions in comparison with conventional Al CC. Similar redox peaks are seen with Mg CC although they are shifted outward corresponding exclusively to the characteristic Na^+^ insertion/extraction processes of the Na_0_._44_MnO_2_ (NMO). These dQ/dV profiles possess multiple redox peaks labeled as 1’, and 2’ during charging, and their complementary peaks 1, and 2 during discharging can be easily identified. These peaks correspond to different reversible phase transitions in the NMO structure caused by sodium‐ion insertion and extraction at tunnel‐type Na2 sites [32]. The charging peaks reflect sequential Na^+^ extraction processes accompanied by the oxidation of manganese ions (Mn^3^^+^ → Mn^4^^+^), while the discharging peaks correspond to the reverse process (Mn^4^^+^ → Mn^3^^+^) during Na^+^ reinsertion [32, 33]. Importantly, no additional redox peaks are observed for either Mg or Mo current collectors when compared to the conventional Al CC system. The absence of additional electrochemical features confirms that no obvious parasitic redox features were detected under the tested conditions. Therefore, the observed performance differences between Mg and Mo CC are primarily attributed to the formation of poorly conductive Mg(OH)_2_/MgO surface layers on Mg, as confirmed by XPS analysis, which increases interfacial resistance rather than inducing unintended electrochemical reactions.

Moreover, from the obtained dQ/dV curves, the Mo CC based battery reveal higher redox peak intensities and a larger integrated area under the curve compared to Mg CC based battery. This enhanced electrochemical activity suggests a superior charge‐storability, likely due to optimized sodium‐ion pathways and reduced charge‐transfer resistance [41]. It can also be noticed that the electrochemical behavior of the present system corresponds to a battery‐type intercalation mechanism rather than a surface‐dominated capacitive process, suggesting that the porosity of the electrodes is very low and negligible. The GCD curves recorded at different C‐rates given in Figure 5c and d demonstrate a gradual decrease in capacity with increasing current density, which is a typical characteristic of battery behaviors. However, Mo CC based battery maintains a higher capacity retention across all rates, as further confirmed by the rate performance test shown in Figure 5e. Even after subjecting the batteries to higher C‐rates up to 4C, Mo CC based battery successfully recovers its capacity when cycled back at C/2, indicating good reversibility. Long‐term cycling stability at a 2C rate (Figure 5f) further validates the enhanced durability of Mo CC, which retains a much higher capacity, 86% of initial capacity over 100 cycles, compared to Mg CC based battery, 76% of initial capacity, which experiences gradual degradation due to oxidation.

Electrochemical impedance spectroscopy (EIS) analysis shown in Figure 5g reveals that Mo CC based battery has a lower series (R1) and charge transfer resistance (R2) of R1:2 and R2: 44 Ω than Mg CC (R1:13 and R2: 52 Ω), contributing to better ionic transport and improved electrochemical performance. Here, R1 mainly reflects the intrinsic ohmic contributions of the cell, including current collector/electrode contact resistance and ionic resistance of the electrolyte, while R2 is associated with charge‐transfer processes occurring at the electrode/electrolyte interface. The lower R1 and R2 values observed for the Mo current collector indicate improved electrical contact at the current collector/electrode interface and more efficient interfacial charge transfer, consistent with the predominantly metallic state of Mo compared to the oxidized Mg layer. Such behaviors are consistent with the XPS depth profiling, which shows that the Mg CC is fully oxidized while the Mo CC is mostly metallic. The EIS spectra obtained after 100 cycles (Figure 5g) show that Mg CC undergoes a noticeable increase in impedance, whereas Mo CC maintains relatively stable resistance, confirming its superior long‐term stability. Overall, these findings suggest that Mo CC is a more promising current collector material than Mg, offering higher capacity, better rate performance, improved cycling stability, and lower internal resistance for efficient energy storage applications. Although the Mg current collector was deposited with a slightly larger thickness (∼600 nm) than Mo (∼400 nm) due to thermal evaporation constraints, EIS measurements (Figure 5g) confirm that the observed electrochemical differences are not governed by thickness‐related resistance but rather by the chemical state of the collectors.

Beyond the general structural degradation mechanisms discussed above, cycling‐related interfacial contact degradation may also contribute to performance decay. This effect is expected to be more pronounced for Mg‐based current collectors due to rapid oxidation and the formation of poorly conductive Mg(OH)_2_/MgO layers, which progressively increase interfacial resistance during cycling. In contrast, Mo current collectors remain predominantly metallic, thereby preserving electrical continuity and stable electrode/current‐collector and electrode/electrolyte interfaces, as supported by the relatively stable impedance evolution observed after prolonged cycling (Figure 5g,h).

To further validate this observed effect, the electronic conductivity of anode and cathode electrodes with Mg/Mo‐CC was carried out before and after the electrochemical cycling using four‐probe conductivity measurements. The results shown in Figure S2 indicate that electrodes (NMO and NTP‐C) with Mg‐CC exhibit relatively lower conductivity than Mo‐CC even before cycling, which can be attributed to the presence of a partially oxidized surface layer, as confirmed by XPS analysis. After cycling, the conductivity of Mg decreased dramatically, while Mo tended to retain a significant portion of its initial conductivity. Specifically, both types of electrodes with Mg‐CC and Mo‐CC exhibited high electrical conductivity prior to cycling, on the order of ∼10^5^ S·m^−^^1^. However, after cycling, a significant difference was observed as electrodes with Mo‐CC maintained relatively high conductivity (∼10^4^ S·m^−^^1^), whereas Mg counterparts showed a substantial decrease in conductivity by several orders of magnitude to ∼10^1^ S·m^−^^1^.

This pronounced reduction in conductivity for Mg is attributed to the formation of MgO and Mg(OH)_2_ surface layers during cycling. The growth of this insulating layer acts as an electron‐blocking barrier, reducing the effective electronic contact between the current collector and the electrode material. Consequently, electron transport across the electrode/current collector interface becomes hindered, leading to increased interfacial resistance and impaired charge‐transfer kinetics. This interpretation is further supported by the electrochemical impedance spectroscopy (EIS) results (Figure 5h), where the Mg‐based system exhibits significantly higher impedance after cycling compared to the Mo‐based system. In contrast, Mo‐CC showed minimal oxidation and maintained a continuous conductive pathway, resulting in improved electron transport and more stable electrochemical performance. This observation supports the formation of an insulating oxide layer on Mg, which acts as an electron‐blocking barrier and contributes to the reduced electrochemical performance observed in Mg‐based systems.

Cross‐sectional SEM analyses before and after electrochemical cycling (ionic) were conducted to investigate the interfacial stability of the electrode/current collector interface. The results show that the interface remains well preserved after prolonged cycling, with no observable delamination, cracking, or void formation. This morphological stability indicates strong interfacial adhesion and suggests that the increase in impedance observed in the EIS data is not associated with structural degradation at the interface. Instead, the impedance increase is more likely attributed to interfacial chemical changes, such as oxide layer formation, rather than mechanical degradation or delamination of the current collector.

To assess the evolution of Mg and Mo CCs upon cycling, XPS depth profiles have been recorded after 100 charge‐discharge cycles. Figures 6 and 7 present the results of this investigation, performed with conditions identical to those described in section 3.1.

**FIGURE 6:**
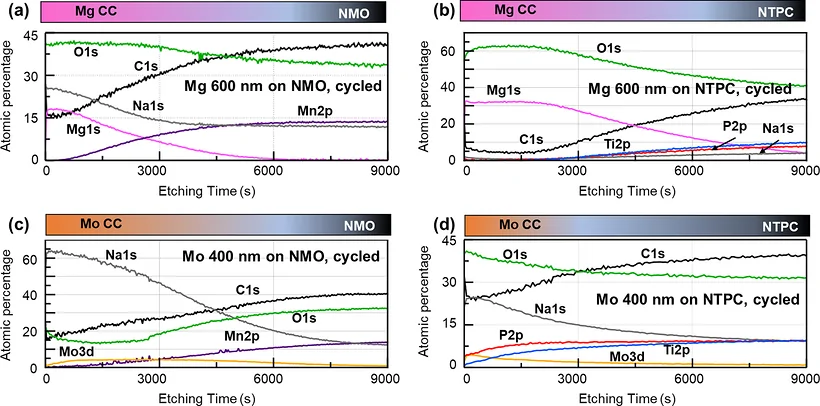
Post‐cycling XPS concentration profiles of (a) Mg CC on NMO, (b) Mg CC on NTPC, (c) Mo CC on NMO, and (d) Mo CC on NTPC. The variation of Mg fraction is represented in pink, Mo fraction in orange, Na fraction in gray, Mn fraction in purple, Ti fraction in blue, P fraction in red, O fraction in green, and C fraction in black. Above each profile is a visual guide (pink represents the Mg CC, orange the Mo CC, black is the electrode, and gray areas correspond to the transition region).

**FIGURE 7:**
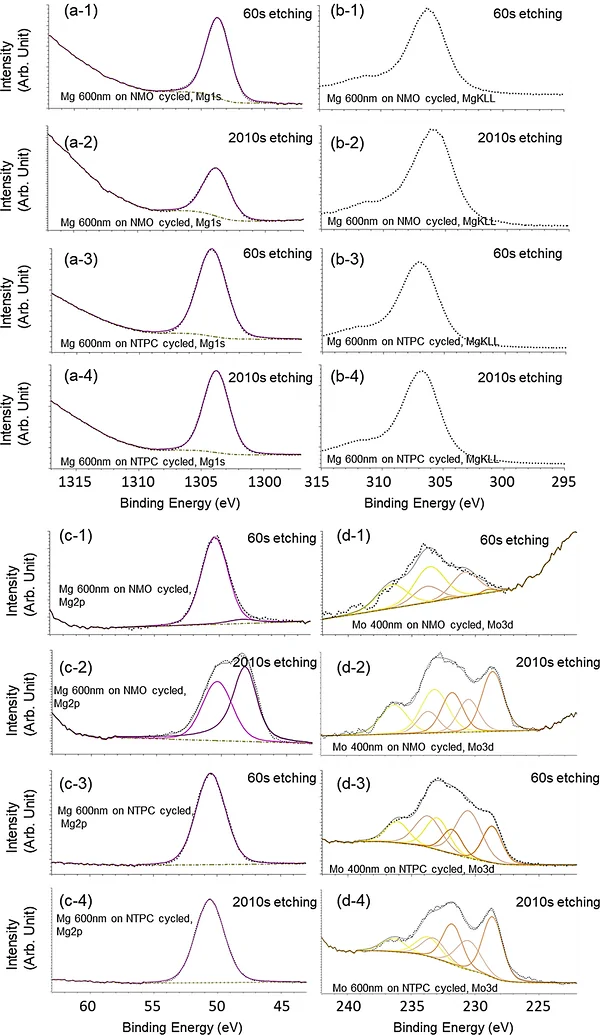
XPS spectra of Mg and Mo current collectors on NMO and NTP‐C, after cycling at the initial stage of the depth profiles (30 or 60 s) and in the bulk of the current collectors (2010 s), (a‐1) Mg 1s peak on NMO after 60 s etching, (a‐2) Mg 1s peak on NMO after 2010 s etching, (a‐3) Mg 1s peak on NTPC after 60 s etching, (a‐4) Mg 1s peak on NTPC after 2010 s etching; (b‐1) Mg KLL peak on NMO after 60 s etching, (b‐2) Mg KLL peak on NMO after 2010 s etching, (b‐3) Mg KLL peak on NTPC after 60 s etching, (b‐4) Mg KLL peak on NTPC after 2010 s etching; (c‐1) Mg 2p peak on NMO after 60 s etching, (c‐2) Mg 2p peak on NMO after 2010 s etching, (c‐3) Mg 2p peak on NTPC after 60 s etching, (c‐4) Mg 2p peak on NTPC after 2010 s etching; (d‐1) Mo 3d peak on NMO after 60 s etching, (d‐2) Mo 3d peak on NMO after 2010 s etching, (d‐3) Mo 3d peak on NTPC after 60 s etching, (d‐4) Mo 3d peak on NTPC after 2010 s etching. Experimental data is shown as a dotted line, Mg 1s and Mg 2p as pink lines, Mn 3p as purple lines, the Mo 3d is decomposed into 3 doublets: Mo^6+^(232.0–235.2 eV) in yellow, Mo^4+^ (229.4–232.6 eV) in light orange and Mo^0^ (228.2–231.4 eV) in dark orange; the Shirley‐type background is presented as a brown dash‐dot line.

Compared with the data obtained before cycling, these results show that i‐there is more intermixing between the electrode materials and the current collectors, especially in the case of Mo CCs; ii‐ the Mg CCs remain totally oxidized and iii‐ the Mo CCs are systematically partially oxidized but a significant part of Mo remains metallic, as shown by the doublets at 228.2–231.4 eV observed in Figure 7d‐1–d‐4.

A summary of various biodegradable/bioresorbable batteries and their main characteristics, including results of the present study are reported in Table 1.

**TABLE 1:** Description and main performance of biodegradable/bioresorbable batteries.

Anode vs. Cathode	Electrolyte	Encapsulation	Operating potential [V]	Capacity [mAh cm^−2^]	Energy density [mWh cm^−2^]	Main feature	References
Mg vs. MoO_3_	Sodium alginate with phosphates	Polyanhydride and PLGA	1.6	6.5	7.15	Bioresorbable	[34]
Mg vs. iodine dispersed in carbon black	Choline chloride/urea	Polyanhydride	1.8	9.8	17.7	Bioresorbable	[35]
Mg vs. Fe	PBS	PCL	0.7	NA	NA	Bioresorbable	[23]
AZ31 vs. Au‐SF	SF‐[Ch][NO_3_]	SF	1.4	0.06	0.078	Biodegradable	[36]
AC vs. λ‐MnO_2_	1 M Na_2_SO_4_	Gelatin	0.6	407	NA	Biodegradable	[37]
Mg vs. Mo	PBS	PDMS	0.75	2.4	1.8	Biodegradable	[28]
Zn vs. α‐MnO2/rGO	CAG with 2 M ZnSO_4_, 0.1 M MnSO_4_	Plasticized silk Protein pocket	0.85‐1.9	211.5 mAh g^−1^	NA	Biodegradable	[38]
AZ31 vs. SF ‐PPy	PBS	NA	1.29	4.42	4.70	Partially biodegradable	[39]
Melanin vs. λ‐MnO_2_	1 M Na_2_SO_4_	NA	1.03	NA	NA	Biocompatible	[40]
Na_2_VTi(PO_4_)_3_ vs. Na_2_VTi(PO_4_)_3_	HydroQSE with 0.5 mol L^−1^ of NaCl	SF hydrogel	1.5	NA	NA	Biocompatible	[41]
NTP & synthetic melanin	Na_2_SO_4_	NA	0.5	78.7	NA	Biocompatible	[42]
Polydopamine/ polypyrrole vs. MnO_2_	Body fluid	Chitosan	1.2	NA	NA	Bioresorbable	[43]
NTP‐C vs. NMO	Na_2_SO_4_ and sodium alginate gel electrolyte	PLGA	1.5	5.05	3.0	Bioresorbable	[10]
Zinc foil vs. α‐MnO_2_	Guar gum/ gelatin/ Zn^2+^(GG‐Zn) hydrogel QSE	SF	0.85‐1.9	292 mAh g^−1^	287 mWh g^−1^	Bioresorbable	[44]
NTP‐C vs. NMO	Na_2_SO_4_ and sodium CMC gel electrolyte	PLGA	1.5	6.8	6.12	Bioresorbable	This work

Abbreviations: NA, not available; PCL, polycaprolactone; PBS, phosphate‐buffered saline; SF –Ppy, silk fibroin‐polypyrrole; SF‐[Ch][NO_3_], silk fibroin−choline nitrate; Au‐SF, gold deposited silk film; AZ31, Mg alloy with 96% Mg, 3% Al, and 1% Zn; Hb, human hemoglobin; QSE, Quasi‐solid‐state electrolyte; CAG, cellulose aerogel‐gelatin.

### 2.3 Biological Studies

#### 2.3.1 In Vitro Tests

To evaluate in vitro cytotoxicity, MTT assay was performed with 6 test samples: Mg CC/NTP‐C/QSE, Mg CC/NMO/QSE, Mg CC/full cell, Mo CC/NTP‐C/QSE, Mo CC/NMO/QSE, Mo CC/full cell for 3 different weight concentrations of 0.075, 0.0375, and 0.01875 g mL^−1^. Figure 8 shows the results of this evaluation. For each test material and at each concentration, it is clear that the cell viability percentage exceeds the 70% threshold red line), suggesting that all test materials are non‐cytotoxic allowing to proceed towards in vivo toxicity studies. It should be noted that the circulatory system in the body is not taken into consideration in in vitro conditions. In vivo circulation system may effectively dilute degradation products. As a result, one of the key differences between in vitro and in vivo toxicity studies is osmolality. Increasing the concentration in vitro, also increases ultimately the osmolality which has been reported as a source of decrease in cell viability [45] and may induce potential osmotic shock to cells, leading to DNA damage [46]. Accordingly, test conditions to osmolality. Accordingly, the lower cell viability values observed under the 0.075 g mL^−^^1^ test condition are attributed to increased osmolality. The toxicity analyses were therefore pursued in in vivo conditions for validation.

**FIGURE 8:**
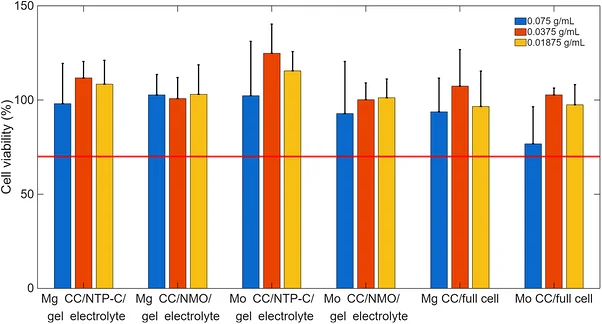
Cytotoxicity evaluation of Mg CC and Mo CC based batteries performed with MTT assay. For each condition, the protocol was repeated 5 times with 2 replicates per protocol (n = 10). The red line denotes the 70% threshold to assess cytotoxicity. Data are represented as mean ± SD. Respectively for 0.075, 0.0375 and 0.01875 g mL^−1^: MgCC/NTP‐C/QSE = 98.1 ± 21.3, 111.8 ± 8.7, 108.5 ± 12.6. MgCC/NMO/QSE = 102.7 ± 10.9, 100.8 ± 11.1, 103.0 ± 15.7. MoCC/NTP‐C/QSE = 102.3 ± 28.8, 124.9 ± 15.4, 115.5 ± 10.3. MoCC/NMO/QSE = 92.8 ± 27.6, 100.2 ± 8.8, 101.3 ± 9.8. MgCC/full cell = 93.7 ± 17.9, 107.4 ± 19.4, 96.6 ± 18.8. MoCC/full cell = 76.7 ± 19.7, 102.7 ± 3.6, 97.5 ± 10.7.

#### 2.3.2 Disintegration Study of Batteries in Biological Media

The bioresorbable Mo CC based batteries was first immersed in a PBS solution at 37°C to monitor its disintegration progress under in vitro conditions as shown in Figure 9. It is clear that the degradation occurs very rapidly; the battery is nearly damaged immediately (Figure 9b) and is fully disintegrated after only 1 h (Figure 9d) and settled down the bottom of the bottle. The increase in the lifespan of the bioresorbable battery during implantable application, can be easily controlled by adjusting the thickness of the soluble encapsulation layer of PLGA, widely used in medical applications due to its biocompatibility and ability to degrade through hydrolysis in biological media. Thus, encapsulation of the batteries with various thicknesses of PLGA layers was achieved by dip‐coating technique. The in vitro disintegration of PLGA‐coated batteries was examined in a PBS solution (static solution). As shown in Figure 9e–h, a battery coated with a 50 µm thick PLGA layer fully disintegrates after 16 days. In contrast, a battery covered with a 500 µm thick PLGA layer takes 90 days to disintegrate (Figure 9i–l). These findings demonstrate that PLGA can significantly delay the degradation process, and the thicker the PLGA layer, the longer the disintegration time.

**FIGURE 9:**
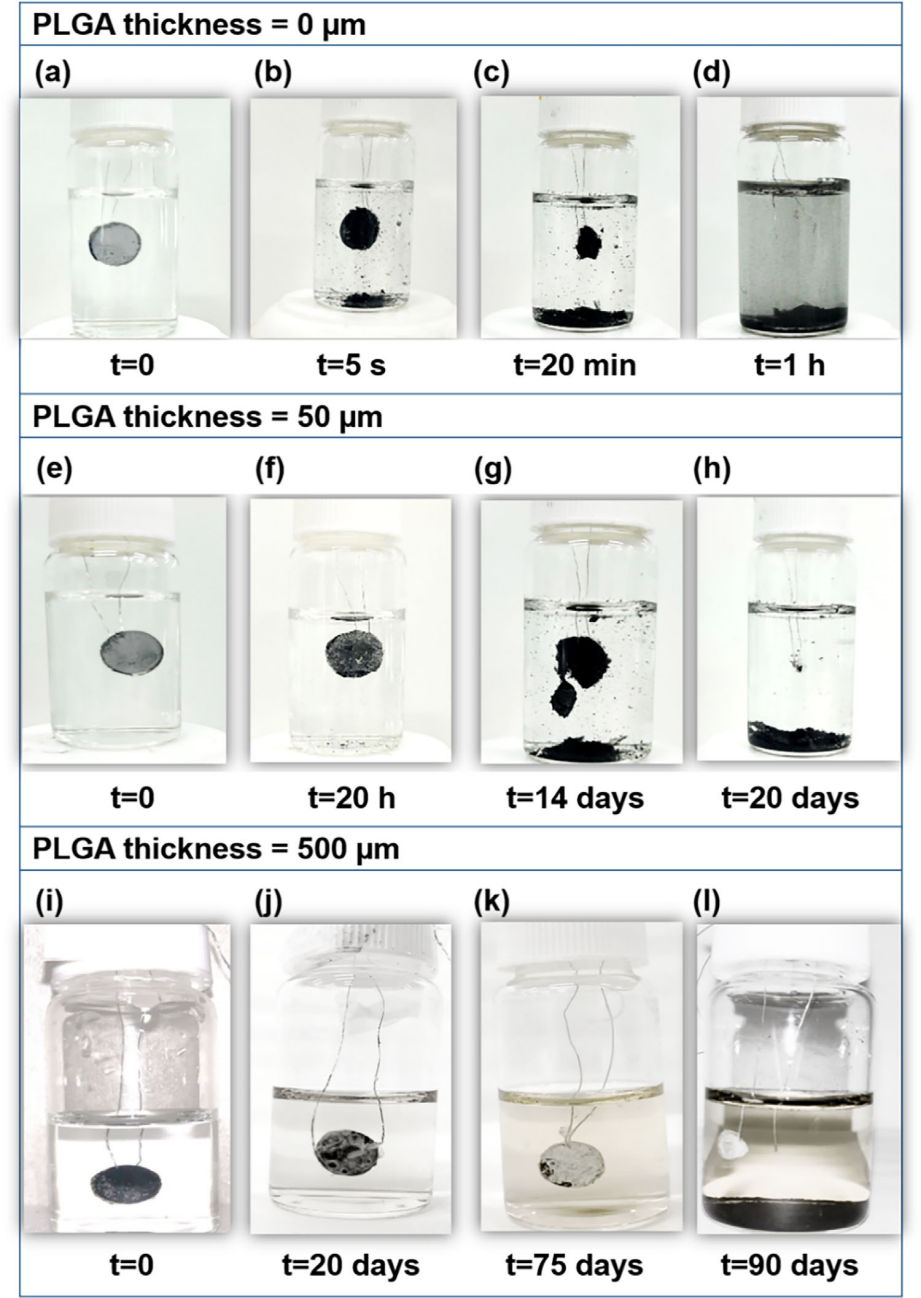
Disintegration evolution of Mo CC based batteries in a PBS solution at 37°C with: (a–d) no PLGA; (e–h) 50 µm thick PLGA layer and (i–l) 500 µm thick PLGA layer.

#### 2.3.3 In Vivo and Ex Vivo Testing: Biocompatibility and Biodegradability of Battery General Integrity Parameters, Weight, and Body Temperature

The animal distribution is reported in Table 2.

**TABLE 2:** Distribution (%) of the 15 mice used in this experiment.

Group	N	%
Female (F)	8 (4B +4S)	53.3
Male (M)	7 (3B +4S)	46.7
Battery (B)	7 (4F+3 M)	46.7
Sham (S)	8 (4F+4 M)	53.3

To evaluate the in vivo biocompatibility of the battery, several general integrity parameters, those that are typically the first to respond to toxicity, were monitored. These included macroscopic observations such as activity levels, tremors, lacrimation, eyelid condition, fur texture, whisker movements, and defecation patterns. Over the course of three months following implantation, no signs of toxicity or adverse changes in the animals' conditions were detected (Table 3).

**TABLE 3:** Score table of SHIRPA Test comparing sham mice (*n* = 8) and battery‐implanted mice (*n* = 7).

	Sham	Battery
**Activity** 0 (inactive) 1 (active) 2 (hyperactive)	1	1
**Tremor** 0 (absent) 1 (present)	0	0
**Lacrimation** 0 (absent) 1 (present)	0	0
**Eyelid closure** 0 (open) 1 (close)	0	0
**Fur** 0 (normal) 1 (bristly)	0	0
**Vibrissae** 0 (present) 1 (absent)	0	0
**Defecation** 0 (present) 1 (absent)	0	0

Body temperature and weight were also monitored in the general health assessment. Body weight gain was monitored over time to evaluate the effect of battery implantation (Figure 10a). While there was no significant effect of implant type (*p* = 0.175) or time (*p* = 0.777), significant interactions were observed between weight and sex (*p* < 0.001) and between weight and implant type over time (*p* = 0.021), suggesting that body weight changes varied across the observation period and were influenced by both sex and implantation (Figure 10a).

**FIGURE 10:**
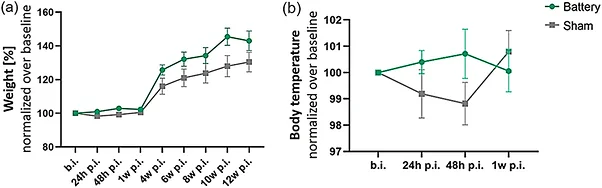
(a) Weight changes over time for all experimental groups, presented as mean ± SEM. Type of implant F (1,12) = 2.079, *p* = 0.175; Sex F (1,12) = 55.799, *p* < 0.001; Time F (8,96) = 0.598, *p* = 0.777; Time*Sex F (8,96) = 10.910, *p* < 0.001; Time*type of implant F (8,96) = 2.393, *p* = 0.021. (b) Body temperature variations over time for all experimental groups, shown as mean ± SEM. Type of implant F (1,12) = 9.605, *p* = 0.009; Sex F (1,12) = 47.918, *p* < 0.01; Time F (3,36) = 1.332, *p* = 0.279; Time*Sex F (3,36) = 1.642, *p* = 0.197; Time*type of implant F (3,36) = 1.221, *p* = 0.316.

Body weight progressively increased in all groups throughout the follow‐up period, indicating normal growth and absence of severe systemic toxicity after implantation. Male animals showed higher baseline and final body weights compared with females, with a mean increase of 18.0 g (42.7%) versus 9.1 g (30.7%), respectively (Table 4). Sex‐related differences were evident, with males displaying higher baseline and final body weights, consistent with expected sexual dimorphism in murine growth patterns. Animals implanted with a battery exhibited a greater weight gain (43.0%) compared with sham controls (30.4%) (Table 4), suggesting that the implant does not impair physiological development and may induce mild metabolic or inflammatory adaptations associated with tissue integration. The significant time‐by‐implant interaction suggests that implant presence modulates growth trajectories rather than causing acute adverse effects. These results indicate that battery implantation affects weight trajectories differently in male and female mice, but does not do so when considered together.

**TABLE 4:** Descriptive statistics (body weight, g) with sex included as a biological variable.

Group	Base weight [g]	12w weight [g]	Δ average weight [g]	% increase
Female (*n* = 8)	29.89	39.01	+9.13	+30.7
Male (*n* = 7)	41.84	59.86	+18.01	+42.7
Battery (*n* = 7)	35.23	50.59	+15.36	+43.0
Sham (*n* = 8)	35.68	47.13	+11.45	+30.4

Body temperature was monitored to assess potential acute inflammatory responses following implantation (Figure 10b). There was a significant main effect of implant type on body temperature (*p* = 0.009), and females had significantly higher temperatures than males (p < 0.01), but time had no significant effect (*p* = 0.279), suggesting that body temperature remained stable across the measured intervals. Furthermore, no significant interactions were observed between time and sex (*p* = 0.197) or between time and implant type over time (p = 0.315), indicating that neither sex nor implantation influenced temperature changes (Figure 10b). Details are available in Tables 5 and 6.

**TABLE 5:** Descriptive statistics (body temperature, *T* °C). Data are presented as mean ± SEM. Difference of body temperature in gender between the two experimental groups.

Male
Time	Battery M (*n* = 3) [*T*°C]	Sham M (*n* = 4) [*T*°C]	*p*‐Value	Significance
Baseline	37.03 ± 0.43	36.83 ± 0.18	0.69	ns
24h	37.27 ± 0.15	36.65 ± 0.25	0.12	ns
48h	37.57 ± 0.17	37.15 ± 0.19	0.09	trend
1week	36.87 ± 0.20	36.65 ± 0.21	0.46	ns

Female
Time	Battery F (*n* = 4) [*T*°C]	Sham F (*n* = 4) [*T*°C]	*p*‐Value	Significance
Baseline	37.68 ± 0.23	38.08 ± 0.19	0.20	ns
24h	37.75 ± 0.15	37.48 ± 0.19	0.26	ns
48h	37.73 ± 0.43	37.40 ± 0.13	0.53	ns
1week	37.83 ± 0.18	37.15 ± 0.24	0.07	trend

**TABLE 6:** Descriptive statistics 2 (body temperature, °C). Data are presented as mean ± SEM. Difference of body temperature between genders within the same experimental group.

Battery group				
Time	Male (*n* = 3) [*T*°C]	Female (*n* = 4) [*T*°C]	*p*‐Value	Significance
Baseline	37.03 ± 0.43	37.68 ± 0.23	0.22	ns
24h	37.27 ± 0.15	37.75 ± 0.15	0.07	trend
48h	37.57 ± 0.17	37.73 ± 0.43	0.70	ns
1 week	36.87 ± 0.20	37.83 ± 0.18	0.015	*

Sham group				
Time	Male (*n* = 4) [*T*°C]	Female (*n* = 4) [*T*°C]	*p*‐Value	Significance
Baseline	36.83 ± 0.18	38.08 ± 0.19	0.003	**
24h	36.65 ± 0.25	37.48 ± 0.19	0.039	*
48h	37.15 ± 0.19	37.40 ± 0.13	0.32	ns
1 week	36.65 ± 0.21	37.15 ± 0.24	0.18	ns

Body temperature did not differ significantly between Battery and Sham groups at any time point in either sex. In males, a non‐significant trend toward higher temperature in the Battery group was observed at 48 h post‐implantation (*p* = 0.09), while no differences were detected at baseline, 24 h, or 1 week. In females, temperatures were comparable between groups at baseline, 24 and 48 h; however, a trend toward higher temperature in Battery animals was observed at 1 week (*p* = 0.07). Overall, battery implantation did not induce statistically significant alterations in core body temperature within the observed period.

Sex‐based differences in body temperature were analyzed within each experimental group at all time points. In the Battery group, no significant sex differences were observed at baseline or 48 h post‐implantation. A trend toward higher temperatures in females compared to males was detected at 24 h (*p* = 0.07). Notably, at 1‐week post‐implantation, females exhibited significantly higher body temperature than males (37.83 ± 0.18°C vs. 36.87 ± 0.20°C, *p* = 0.015). In the Sham group, females displayed significantly higher baseline body temperature compared to males (38.08 ± 0.19°C vs. 36.83 ± 0.18°C, *p* = 0.003). This difference remained significant at 24 h (37.48 ± 0.19°C vs. 36.65 ± 0.25°C, *p* = 0.039). However, no sex‐related differences were detected at 48 h or 1 week. These results indicate a physiological sex‐related difference in baseline temperature in sham‐operated animals, whereas in the Battery group a significant sex divergence emerged only at one‐week post‐implantation. Overall, these findings on body weight and temperature support good systemic biocompatibility, with no detectable adverse response following implantation.

#### 2.3.4 Behavioral Testing: Systemic Toxicity Assessment

Systemic toxicity was further assessed using the Open Field (OF) behavioral test to measure locomotion and to evaluate anxiety levels, as these are the initial behaviors that are known to change in cases of systemic toxicity. Evaluations were conducted before the implant (baseline) and at 1, 2, and 3 months after surgery.

Results shown in Figure 11 demonstrated that locomotor activity (distance travelled) was influenced by both implant type and sex (*p* = 0.012, *p* < 0.001, respectively), whereas anxiety‐like behavior (% time in center) was primarily affected by sex (*p* = 0.049), with a trend for implant type (*p* = 0.062). Maximum speed was not significantly affected by any factor. These results suggest that sex and implant type contribute to differences in exploratory activity, but anxiety‐like behavior is predominantly sex‐dependent, and no significant alterations were observed in maximum speed. Overall, these results indicate a good biocompatibility of Mo CC based batteries.

**FIGURE 11:**
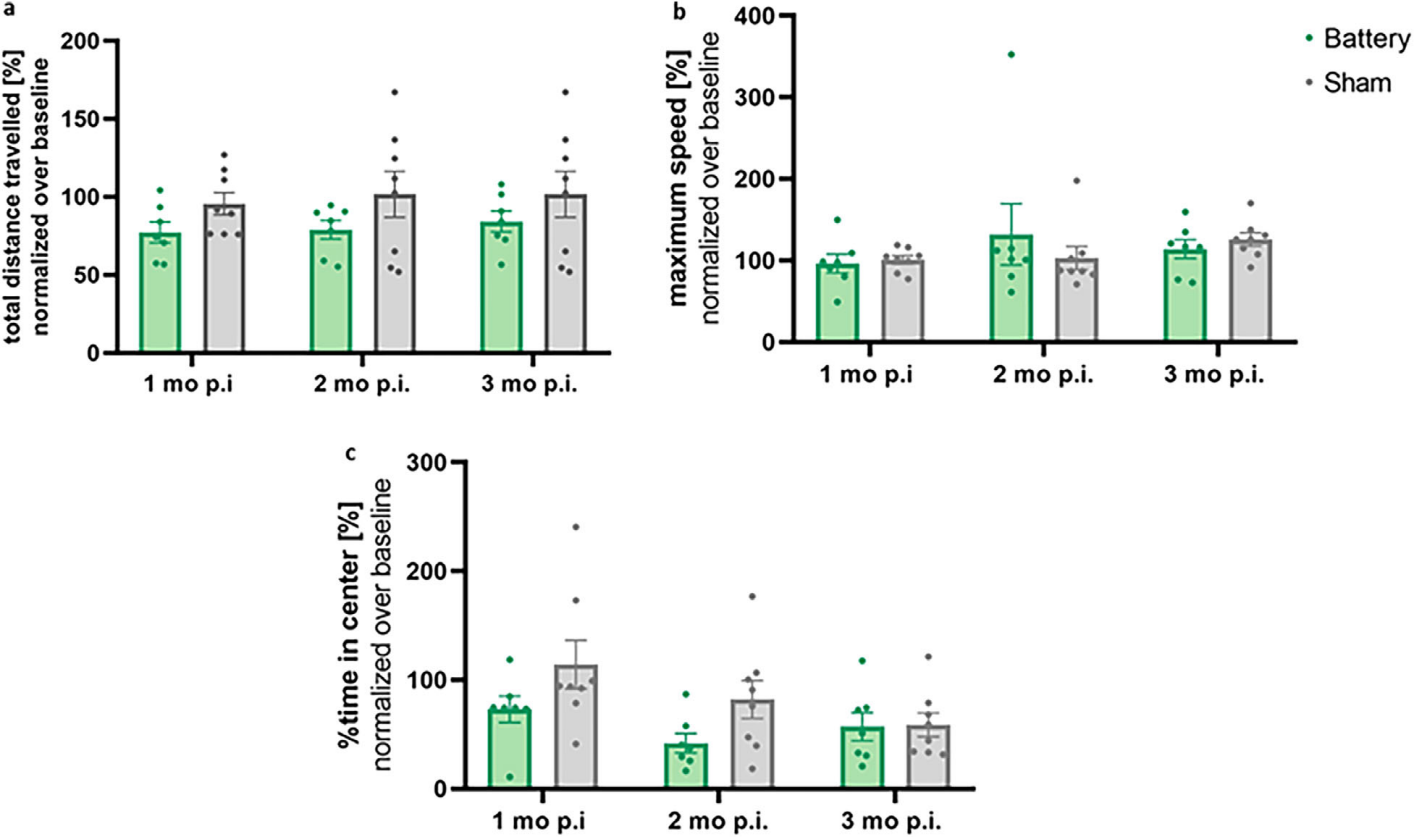
Open Field test: (a) total distance travelled [m], (b) maximum speed [m/s], and (c) percentage of time spent in the center of the arena normalized over baseline (%). All data were represented as mean ± SEM. Statistical analyses were performed using repeated‐measure ANOVA, type of implant as a factor, and sex as covariate. Distance travelled: Type of implant F (1,12) = 8.645, *p* = 0.012; Sex F (1,12) = 22.551, *p* < 0.001; Time F (2,24) = 2.712, *p* = 0.087; Time*Sex F (7,84) = 2.649, *p* = 0.091; Time*type of implant F (2,24) = 0.769, *p* = 0.475. Max speed: Distance travelled: Type of implant F (1,12) = 0.075, *p* = 0.788; Sex F (1,12) = 0.104, *p* = 0.753; Time F (2,24) = 2.046, *p* = 0.151; Time*Sex F (2,24) = 2.007, *p* = 0.156; Time*type of implant F (2,24) = 1.214, *p* = 0.315. % time spent in the center: Type of implant F (1,12) = 4.241, *p* = 0.062; Sex F (1,12) = 4.808, *p* = 0.049; Time F (2,24) = 0.566, *p* = 0.575; Time*Sex F (2,24) = 0.538, *p* = 0.591; Time*type of implant F (2,24) = 1.741, *p* = 0.197.

In detail: behavioral testing revealed reduced locomotor activity in animals implanted with a battery compared with sham controls, with approximately 20% lower travelled distance during follow‐up assessments. This suggests that device implantation may modulate spontaneous exploratory behavior without inducing severe motor impairment. Sex‐dependent differences were evident across behavioral domains. Female mice generally maintained higher locomotor activity and showed greater exploratory behavior, whereas males more frequently exhibited reduced activity relative to baseline. Classification analyses indicated that a higher proportion of males fell into the decreased activity category, while females more often displayed stable or increased behavioral performance. Maximal speed and center‐zone exploration (% time center), used as proxies for motor performance and anxiety‐like behavior, respectively, showed substantial inter‐individual variability but no indication of severe anxiety‐related phenotypes induced by implantation. These findings suggest that behavioral responses to implantation are influenced by sex‐specific physiological adaptations and highlight the importance of including sex as a biological variable in biocompatibility studies.

#### 2.3.5 Organ Inspection: Weight Assessment and Biodegradability

At sacrifice, organs, including the liver, spleen, kidneys, heart, lungs, and brain, were collected and weighed. No significant abnormalities or differences in organ condition were observed between the two groups (Figure 12), confirming the non‐toxic nature of the implanted battery.

**FIGURE 12:**
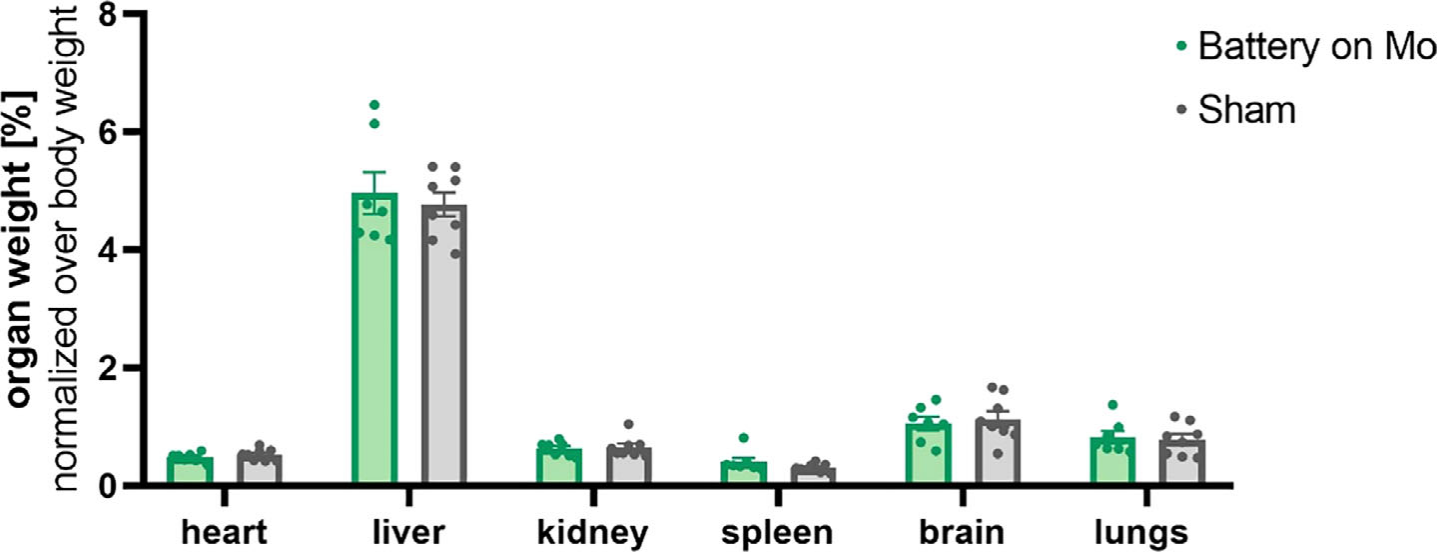
Organ weights at sacrifice. Data presented as mean ± SEM and analyzed by means of repeated measure ANOVA. Type of implant F (1,12) = 0.309, *p* = 0.589; Sex F (1,12) = 3.155, *p* = 0.101; Organ type F (5,60) = 31.286, *p* < 0.001; Organ type*Sex F (5,60) = 20.720, *p* < 0.001; Organ type*type of implant F (5,60) = 1.185, *p* = 0.327.

To evaluate the bioresorbability of battery, the subcutaneous pocket corresponding to the site of implant was opened, revealing complete disappearance of the battery with minor traces of black carbon remaining.

#### 2.3.6 Histological Analysis of Skin

Histological cross sections of the skin were cut on a cryostat into 40 µm slices and colored with Hematoxylin & Eosin. As shown in Figure 13, residues of carbon black are observed in the battery‐implanted animals at the subcutaneous layer, due to the release of black carbon. However, histological evaluation of the layer structure for necrosis or inflammation showed that the skin thickness and layer distribution is not altered compared to sham mice, indicating no adverse local toxicity despite the presence of the deposit.

**FIGURE 13:**
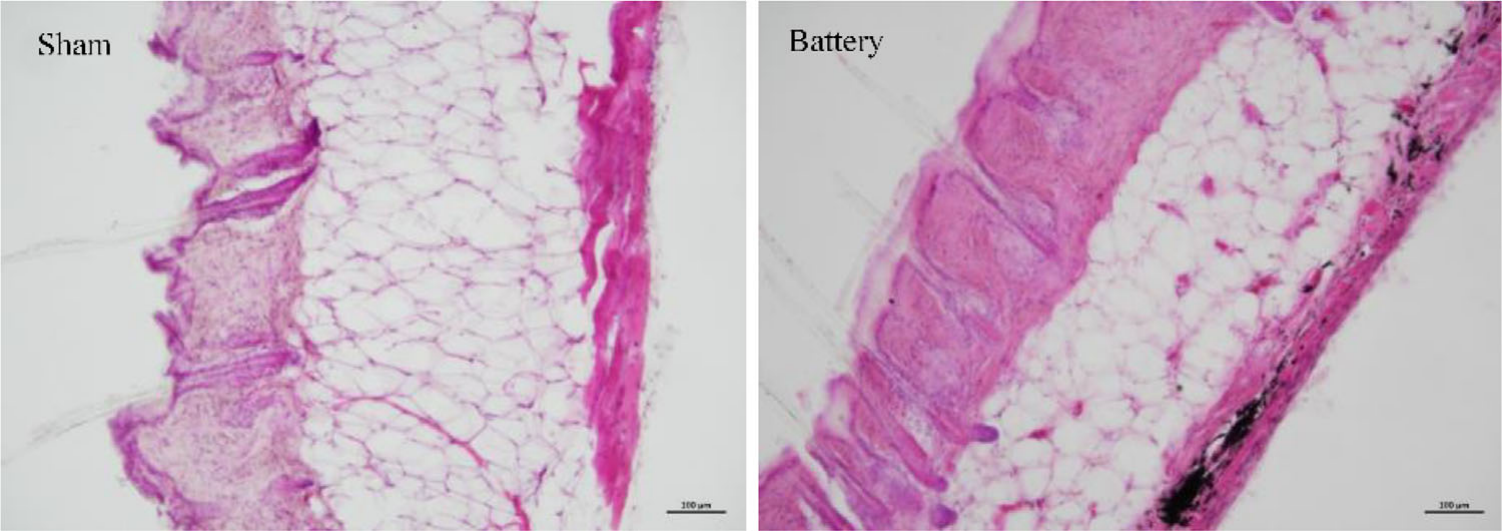
Representative images of hematoxylin and eosin‐stained cross sections of the skin of sham and battery mice. Residues of carbon black are observed as black deposits. Scale bar = 100 µm.

#### 2.3.7 Hepatic ALT Evaluation

Serum alanine aminotransferase (ALT), a key indicator of liver injury and systemic toxicity, was analyzed to confirm the absence of hepatic impairment. Comparable ALT levels were observed across the two experimental groups (Figure 14), supporting the conclusion that battery implantation did not cause liver dysfunction or systemic toxic effects.

**FIGURE 14:**
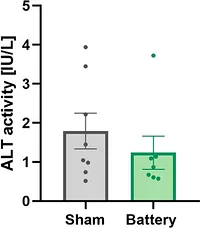
Serum ALT activity (IU/L) in experimental groups (sham *n* = 8; battery *n* = 7) (mean ± SEM). Data analyzed using non‐parametric *t*‐test (*p* < 0.05 considered significant; *p* = 0,3940).

### 2.4 Controlling the Battery Disintegration Time In Vitro

The long‐term electrochemical performance and degradation behavior of bioresorbable batteries encapsulated with PLGA coatings of varying thicknesses ranges from 0, 50 µm, 100, 250, and 500 µm were systematically analyzed at a 1C rate. The 1C rate was selected for disintegration studies to accelerate cycling‐induced degradation and enable clear identification of capacity decay and failure onset within experimentally accessible timeframes, while remaining within a practical operating regime. As shown in Figure 15, the initial discharge capacity remains stable across all samples in the early stages, but degradation begins at different time points depending on the PLGA thickness. The Mo CC based battery without encapsulation rapidly deteriorates, whereas the 50 µm encapsulated battery extends its functional lifetime to 16 days before entering the disintegration zone. Further increasing the PLGA thickness to 100 µm and 250 µm prolongs battery operation to 37 days and 51 days, respectively, highlighting the protective role of the encapsulation layer. The 500 µm encapsulated bioresorbable battery exhibits the longest stability, maintaining its discharge capacity beyond 90 days, demonstrating the most effective encapsulation strategy. The working zone, where the battery retains at least 90% of its initial capacity until 75 days, is significantly extended with thicker PLGA layers, confirming that encapsulation slows down degradation by limiting electrolyte infiltration and structural breakdown. These results indicate that PLGA encapsulation is a key factor in tuning the lifetime of bioresorbable batteries, making them suitable for various implantable bioelectronic applications requiring controlled degradation profiles.

**FIGURE 15:**
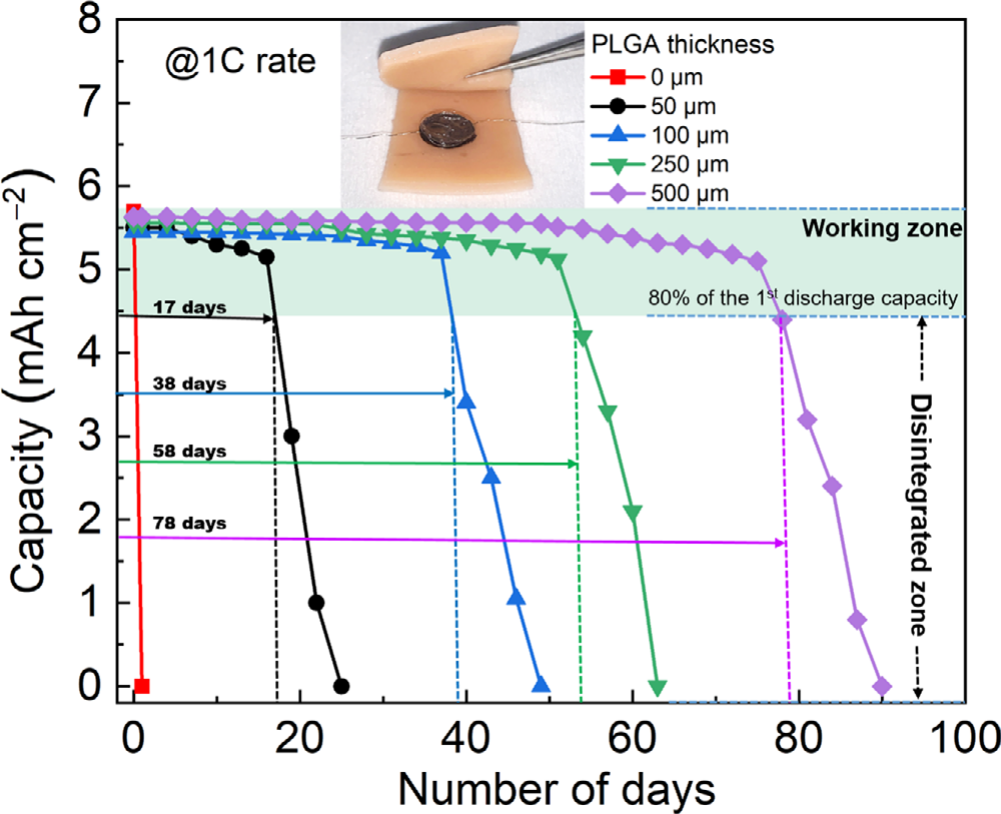
Evolution of the discharge capacity as a function of the operating day for bare and PLGA‐coated batteries implanted in artificial skin.

### 2.5 Electrochemical Performance and Functioning Under Implantation‐Mimicking Conditions

To further assess the functionality of the battery under physiologically relevant conditions, additional electrochemical and operating tests were performed 37°C. As shown in Figure 16a, galvanostatic charge‐discharge measurements at C/10, C/2, and 1C rates exhibited capacities comparable to those obtained at room temperature, indicating that the electrochemical activity of the cell is preserved at physiological temperature. The corresponding differential capacity plot (dQ/dV) recorded at C/10 (Figure 16b) shows well‐defined redox peaks, confirming that the charge‐storage mechanism remains unchanged. Long‐term cycling at 1C (Figure 16c) revealed an initial capacity decrease of approximately 14% during the first 10 cycles, followed by stable operation with approximately 70% capacity retention over the subsequent 100 cycles. Compared to analyses performed at room temperature, this slight capacity loss could be attributed to the faster activation of corrosion processes that are responsible for limiting the electron transfer.

**FIGURE 16:**
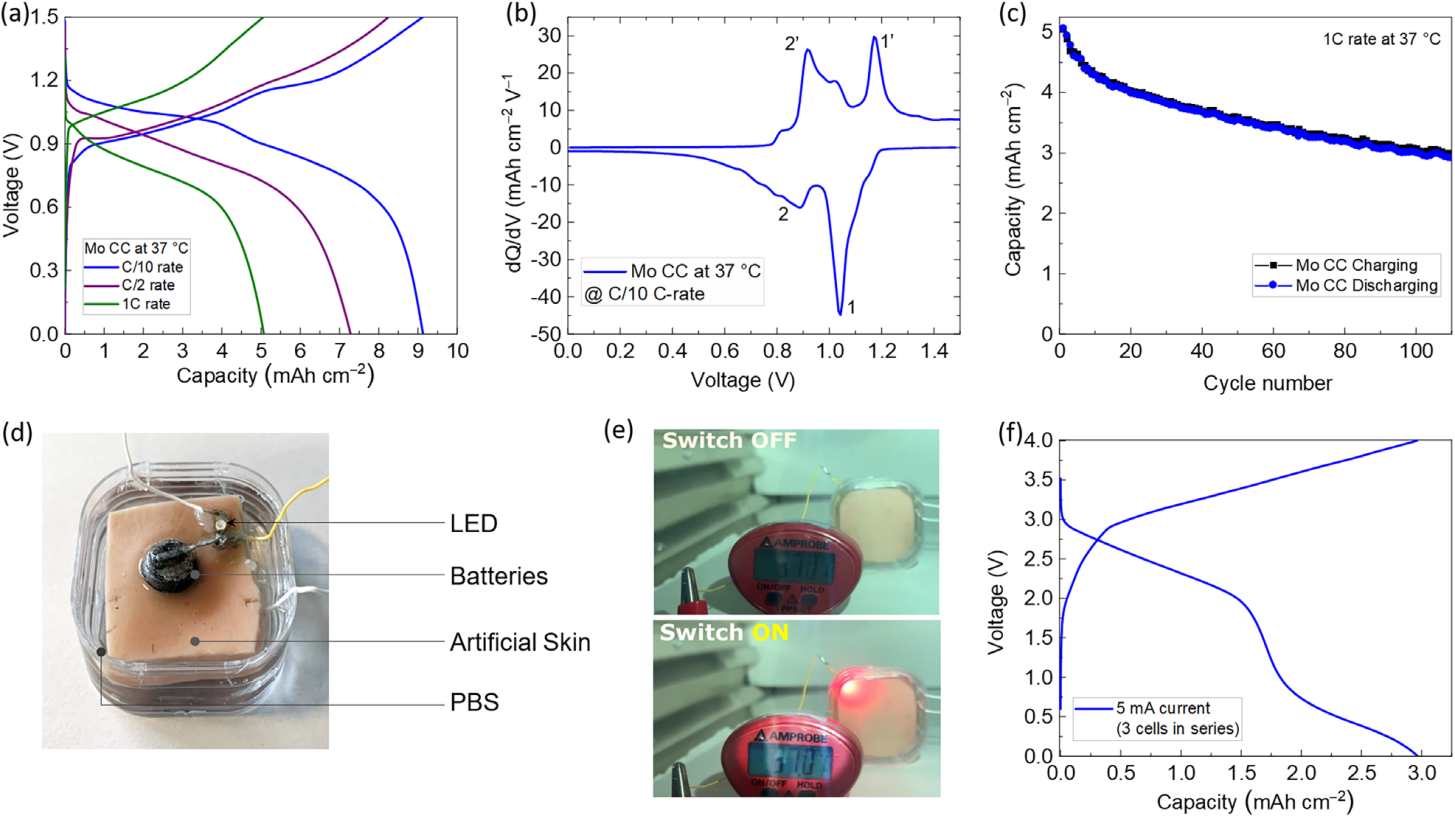
Electrochemical and operating tests of the Mo‐CC bioresorbable sodium‐ion battery under physiologically relevant conditions at 37°C. (a) Galvanostatic charge‐discharge curves measured for C/10, C/2, and 1C rates. (b) dQ/dV profile obtained at C/10. (c) Cycling performance at 1C for 110 cycles. (d) Demonstrator of an encapsulated battery composed of 3 cells placed beneath artificial skin in PBS at 37°C powering an LED. (e) Representative galvanostatic discharge profile of three cells connected in series under a constant current of 5 mA in the tissue‐mimicking environment.

In order to move towards a demonstrator working under simulated in vivo conditions, a functional device was designed and placed beneath artificial skin in phosphate‐buffered saline (PBS) maintained at 37°C. Three cells connected in series were able to continuously power a light‐emitting diode (LED), demonstrating active power delivery under hydrated conditions relevant to implantation (Figure 16d,e and Video S1). A representative galvanostatic discharge profile of the series‐connected cells recorded at a constant current of 5 mA is presented in Figure 16f, further confirming the capability of the battery system to provide stable electrical output in this tissue‐mimicking environment. It can be pointed that cells in series allowed to reach the threshold voltage to power the LED while the current density remained the same as a single cell. Although these experiments do not constitute direct in vivo electrochemical validation, they provide additional evidence supporting the functionality of the bioresorbable sodium‐ion battery under physiologically relevant conditions and bridge the gap between conventional in vitro characterization and future implantation studies.

## 3 Conclusion

In this study, new quasi‐solid bioresorbable Na‐ion batteries were successfully fabricated for temporary medical applications and resorbtronics. The electrochemical performance of the batteries varied with the choice of current collector, with Mo layer offering enhanced stability compared to its Mg thin‐film counterpart. The best system demonstrated a high discharge capacity 6.8 mAh cm^−^^2^ at a C/2 rate, while in vivo studies confirmed the safe bioresorption of the device without toxicity or organ damage. In particular, it is possible to state that the batteries have a good biocompatible, since the parameters evaluated in vivo and *ex vivo*, overall, do not differ from those obtained from the sham group. Moreover, the batteries were shown to be bioresorbable, as no residues were detected at the implantation site after 3 months, with the exception of minor traces of carbon black only in the area in contact with the battery. XPS analysis provided valuable insights into the chemical changes at the Mo and Mg electrodes before cycling, revealing important degradation mechanisms due to the oxidation of Mg. Furthermore, the operational lifetime of these bioresorbable batteries was demonstrated to be finely tuned by adjusting the encapsulation layer thickness, allowing for controlled degradation timelines ranging from days to weeks. Overall, this work presents a promising step toward the development of next‐generation transient power sources for biomedical devices. By combining appealing features like biocompatibility, stable electrochemical performance, and tunable degradation rates, these bioresorbable Na‐ion batteries represent a first promising step towards the powering of functional platforms dedicated to temporary implantable medical devices. While the present study demonstrates stable electrochemical performance of batteries implanted in tissue‐mimicking conditions at 37°C, further battery tests under in vivo conditions, including voltage output, capacity retention, and long‐term performance will have to be investigated in the future to fully validate this proof‐of‐concept.

## 4 Experimental Section

### 4.1 Materials

As active electrode materials, 3 wt.% of carbon‐coated NaTi_2_(PO_4_)_3_ (NTP‐C) used as anode and Na_0.44_MnO_2_ (NMO) used as cathode were purchased from NEI corporation, USA. The sodium carboxymethyl cellulose (Na‐CMC), phosphate buffer saline pellets, poly (lactic‐co‐glycolic acid) (PLGA; lactide: glycolide 65:35; Mw 40,000‐75,000), and Na_2_SO_4_ were purchased from Sigma Aldrich. Carbon black (super P conductive) was used as conductive agent in the electrodes (99+% metal basis, Thermo Fisher Scientific, USA). Mg (Mg, 99.99%) pellets (0.6 mm diameter and 6 mm length), molybdenum (Mo, 99.95%) pellets (3 mm diameter and 3 mm length) and tungsten liners were purchased from Neyco Vacuum & Materials. Artificial human tissues with 2 N skin toughness (SynDaver, USA) was used for disintegration studies. All materials used to fabricate the bioresorbable batteries with Mo current collector (Mo CC) and Mg current collector (Mg CC) are mainly composed of the materials solely of biocompatible whose safety has been verified through the analysis of toxicological files published by the Institut National de Recherche et de Sécurité (INRS).

### 4.2 Electrode Pellets Fabrication

The three main stages of the battery fabrication process are schematically represented in Figure 17 as a stepwise sequence comprising (a) electrode pellet fabrication, (b) electrolyte preparation and full‐cell assembly, and (c) thin‐film metal deposition. The experimental procedures for electrode fabrication, electrolyte preparation, cell assembly, and electrochemical characterization were carried out following the protocols reported in our earlier work [10].

**FIGURE 17:**
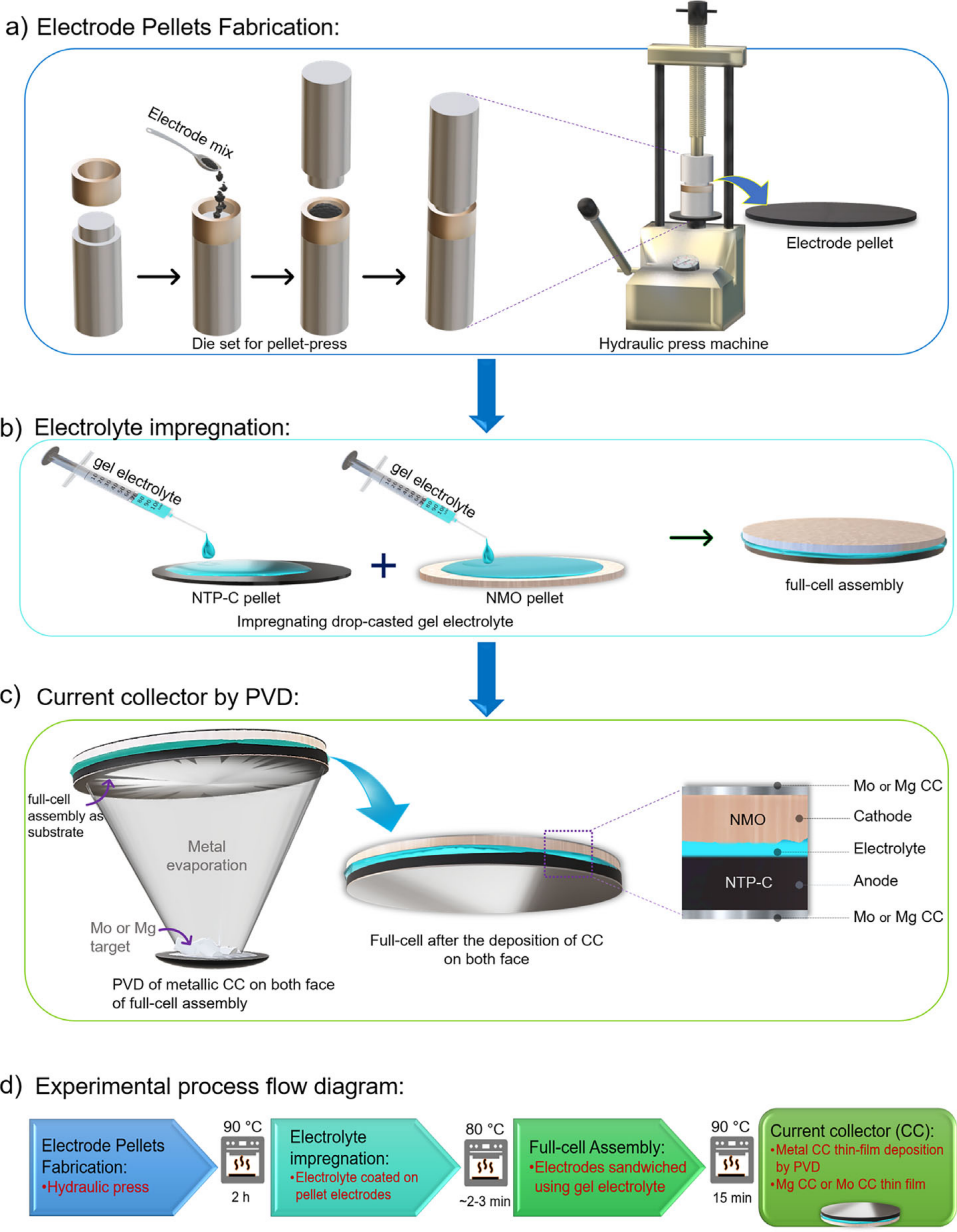
Schematic of the bioresorbable battery fabrication sequence. (a) Formation of pellet electrodes by hydraulic press machine, (b) impregnating drop‐casted gel type electrolyte onto electrode, and assembly of the full cell, and (c) evaporation of metal thin‐film (Mo or Mg) on both remaining electrode faces. (d) Overall experimental workflow diagram of the fabrication process.

Corresponding to Figure 17a, anode (NTP‐C) and cathode (NMO) were fabricated in a pellet form by making a mixture of active materials (80%), conductive carbon (10%), 5% of Na CMC as binder, 5% of electrolyte mixture, and deionized water (DI) as solvent. The powders were mixed into a fine powder with a mortar and pestle, made into a wet form to fill 1 mm inner diameter of dies and pressed the mixture in a hydraulic press machine with an applied pressure of 2 tons cm^−^^2^. The applied pressure of 2 tons cm^−^^2^ was optimized to achieve sufficient electrode densification and particle–particle contact, while higher pressures induce crack formation due to elastic spring‐back after pressure release, ensuring mechanically robust pellets suitable for uniform current collector deposition. The prepared electrode pellets were dried for 2 h in a 90°C vacuum oven.

### 4.3 Preparation of Electrolyte and Full Cell Assembly

Corresponding to Figure 17b, gel type polymer electrolyte was made by dissolving 1.6 g Na_2_SO_4_ in 10 mL DI water and adding 400 mg sodium carboxymethyl cellulose (Na‐CMC) to the solution. A gel‐type electrolyte was employed to ensure good electrode–electrolyte interfacial contact and efficient Na^+^ transport while avoiding rapid electrode disintegration associated with liquid electrolytes. The salt to polymer ratio was maintained as 4:1. This ratio was selected to balance ionic conductivity and mechanical stability of the electrolyte once dried. The mixture was stirred for 1 h to get completely dissolved thick gel type electrolyte. As prepared, gel type polymer electrolyte was coated over cathode and anode electrodes and dried at 80°C for 2 to 3 min to be sure that the electrolyte is completely dried to form Quasi‐solid‐state electrolyte (QSE) as shown in Figure 17b. The electrolyte‐impregnated anode and cathode pellets were sandwiched together by wetting the surfaces with the gel form of the electrolyte and dried 15 min at 90°C. As prepared, full cells were taken for metal evaporation on both surfaces.

### 4.4 Thin Film Metal Deposition

Metal evaporation corresponding to Figure 17c, that is. Mo or Mg, was carried out in the Alliance concept, Eva 450 through Thermal Evaporation‐Joule effect method. Evaporation parameters were set as density 1.740 g cm^−3^ and Z factor 1.610. Metallic pellets were filled in a tungsten liner and started evaporation at a pressure of 5 × 10^−6^ Torr. The outer surface of both electrode pellets was covered by evaporated Mg (∼600 nm) to work as a current collector. Mo thin layer (∼400 nm) deposition was also carried out in same system of Eva 450, through e‐beam method with density 10.20 g cm^−3^ and Z factor 0.257 at a pressure of 5 × 10^−6^ Torr. The slightly larger Mg thickness was required to ensure continuous coverage on the porous pellet substrate during thermal evaporation, whereas e‐beam deposited Mo forms denser films that allow reliable current collection at lower thickness.

### 4.5 Physical and Electrochemical Characterization

CARL ZEIS‐S/Ultra 55 Scanning Electron Microscope (SEM) machine was used to analyze surface and morphology of electrode pellets and biodegradable batteries. The Energy Dispersive X‐Ray Spectroscopy (EDX), associated with SEM was used for the elemental mapping. To obtain the cross‐sectional SEM image of the batteries with Mo and Mg CC before and after electrochemical tests, the battery sample was first embedded in epoxy resin to provide sufficient mechanical support and structural integrity during preparation. After curing, the embedded sample was sequentially polished using sandpapers. This stepwise polishing process allowed us to expose the cross‐section while achieving a smooth and uniform surface and to have high quality SEM images. X‐Ray Photoelectron (XPS) measurements were performed using a Thermo Electron Ka spectrometer equipped with an Al Ka X‐ray Source, a dual (Ar^+^ ions and electrons) flood gun for charge neutralization and an Ar^+^ etching source. Depth profiles were acquired with the following settings for the Ar^+^ etching source (200 to 300 steps, 3000 eV accelerating voltage, High current, step duration of 30s and the spectra were recorded in Snapshot Mode with a Pass Energy of 150 eV during the depth profile. Prior to the depth profiles, survey spectra and high‐resolution windows (not shown here) were recorded in Scan Mode with pass energies of 200 and 20 eV, respectively. The XPS data were treated with CasaXPS [47], Shirley‐type functions were used for the background, and the components were described by Voigt functions to fit the high‐resolution spectra. The galvanostatic charge and discharge characteristics of the batteries were analysed in a two‐electrode system configuration using the VMP3 (Bio Logic) potentiostat‐galvanostat at a constant temperature (298 K).

### 4.6 In Vitro Cytotoxicity

In vitro cytotoxicity tests were made according to the International Organization for Standardization (ISO) guidelines (ISO 10993 parts 5 and 12) [48, 49].

#### 4.6.1 Cell Culture

Mouse Fibroblast cells (ATCC CCL‐1) were purchased from ATCC and cultured at 37°C in a humidified atmosphere with 5% CO_2_. Cells were maintained in ATCC‐formulated Eagle's Minimum Essential Medium (EMEM) supplemented with horse serum (Gibco, Horse Serum ABP‐040) to a final concentration of 10%, 100 units mL^−1^ of penicillin, and 100 µg mL^−1^ of streptomycin (Gibco, 15140‐122).

#### 4.6.2 Extract Preparation

For cytotoxicity assay, 6 different extracts were prepared: cathode/electrolyte, anode/electrolyte, and full battery with either Mg CC of Mo CC deposited on the electrodes. Cathode and anode were immersed in the culture medium described previously for 24 h under cell culture conditions (5% CO_2_, 95% humidity, 37°C) at 1.25 cm^2^ mL^−1^ corresponding to 0.075 g mL^−1^. For better comparison, full batteries were also immersed in the culture medium at 0.075 g mL^−1^. The extracts were then collected filtered at 0.8 µm, pH adjusted, and 3 different dilutions were tested: 1:1, 1:2, and 1:4.

#### 4.6.3 MTT Assay

MTT assay was performed according to ISO guidelines with standard protocol used in the literature [50, 51]. Briefly, L929 cells were seeded on 96‐well plates with a density of 10.000 cell per well and precultured in normal cell culture medium for 24 h for facilitating cell attachment. The medium was then replaced by 100 µL of the extracts for the test samples, fresh culture medium for negative cytotoxicity control or fresh medium with 10% dimethyl sulfoxide (DMSO) for positive cytotoxicity control and incubated for 24 h. 12 mM MTT solution was prepared by adding 5 mg MTT (3‐(4,5‐Dimethylthiazol‐2‐yl)‐2,5‐Diphenyltetrazolium Bromide) into 1 mL PBS prior to filtration and sterilization. 10 µL of this MTT solution was added to each cell after 24 h of incubation with the test samples. Then, cells were incubated for 4 h followed by the removal of 85 µL of medium and addition of 100 µL DMSO into each well to dissolve the formazan crystals. Absorbance measurements were made within an hour at 570 nm and corrected by absorbance measurement at 650 nm using a microplate reader (TECAN Infinite M1000). Experiments were repeated 10 times (The full protocol was reproduced 5 times with 2 replicates per protocol, *n* = 10).

#### 4.6.4 Data Analysis

Outliers were identified using the boxplot interquartile range (IQR) method and were defined as values lying more than 1.5 × IQR below the first quartile or above the third quartile. The percentage of cell viability was calculated with the following equation:

$$Cell\;viability\;\left(\%\right)=\frac{OD_{sample}-OD_{pos\;control}}{OD_{neg\;control}-OD_{pos\;control}}\;X\;100$$

Where *OD*_sample_, *OD*_pos control_ and *OD*_neg control_ were the absorbance of the test sample, the cytotoxic positive control, and cytotoxic negative control conditions respectively

### 4.7 In Vitro Disintegration Assay

Mo CC based batteries were encapsulated with PLGA using various thicknesses and dipped in 10 mM phosphate‐buffered saline (PBS) solution at pH 7.4. To maintain the simulated physiological condition at constant temperature (37°C), tests were performed in a CTS Clima temperature system. To evaluate the electrochemical performance of Mo CC based batteries and degradation behavior under in vitro conditions, the encapsulated batteries were sandwiched using the pre‐soaked (PBS solution) artificial skin sample (SynDaver, ∼3.5 × 2.5 cm, 1.5 mm thick). A battery without encapsulation was also taken for both in vitro disintegration assay in PBS solution and sandwiched within synthetic skin for the comparison studies. Galvanostatic charge‐discharge measurements were conducted at a 1C rate for each battery to assess its electrochemical performance over time. To ensure a stable testing environment, fresh PBS solution was introduced into the setup every two days, maintaining the natural hydration level and consistent wettability of the surrounding tissue. This approach allowed for continuous monitoring of capacity retention, providing critical insights into the onset and progression of battery degradation under physiological conditions.

### 4.8 In Vivo and Ex Vivo Studies

#### 4.8.1 Animals

Three‐month‐old CD1 mice (*n* = 15, 7 male and 8 female) were used for this study. Animals were supplied by Charles River Laboratories Italia s.r.l. (Calco, Milan, Italy). Upon arrival, mice were housed in a specific pathogen‐free facility under controlled environmental conditions (temperature: 21 ± 0.5°C; humidity: 60%) on a 12‐h light/dark cycle, with ad libitum access to food and water. All procedures were approved by the Committee on Animal Health and Care of the University of Modena and Reggio Emilia and were conducted in accordance with the National Institutes of Health guidelines (CEE Regulation 2010/63/EU 2010/63/EU, Italian Legislative Decree 26/2014, authorization no. 979/2020/PR). All efforts were made to minimize animal suffering and reduce the number of animals used in accordance with the Guideline on the principles of regulatory acceptance of 3Rs (Replacement, Reduction, Refinement).

#### 4.8.2 Battery Implantation Procedure

Animals were randomly assigned to two experimental groups with balanced sex distribution: (i) the Sham group (4 M, 4F), subjected to surgical procedures without battery implantation, and (ii) the Battery group (4F, 3 M), receiving a subcutaneous battery implant as shown in Figure 18a.

**FIGURE 18:**
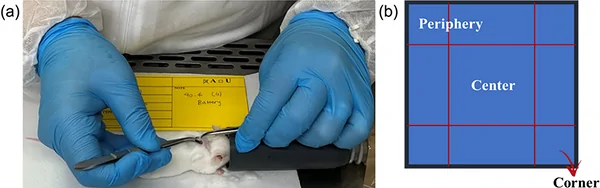
(a) Representative image of battery implantation. (b) Representative image of the OF arena.

Mice were anesthetized with inhaled isoflurane, and the dorsal skin was shaved and disinfected with povidone‐iodine. A small incision was made, and a subcutaneous pocket (1.5 × 1.5 cm) was created to accommodate the battery. The incision was closed with a surgical suture and treated with Neuflan gel (Neomycin 0.5 g / Fluocinolone acetonide 0.025 g / Lidocaine 2.5 g) to minimize postoperative pain and prevent infections.

#### 4.8.3 In Vivo Toxicity and Biodegradability Assessment

Toxicity and biodegradation were evaluated over a 3‐month observational period following surgery, in accordance with UNI EN ISO 10993–6 guidelines. Throughout this period, the general health status of the animals was monitored daily and formally scored at 24 and 48 h, and at 1, 4, 6, 8, 10, and 12 weeks after surgery, according to a modified SHIRPA protocol [52]. Several parameters were analyzed, including gross behavioral and physical trails such as activity, tremor, lacrimation, eyelid closure, fur condition, whisker movement, and defecation, which were scored on a binary scale (0 = absent, 1 = present).

Body weight was recorded throughout the study and normalized to pre‐implantation values. Rectal body temperature was measured before implantation (baseline) and at 24, 48 h and One‐week post‐implantation to exclude acute and sub‐acute inflammatory responses. Locomotor activity and anxiety‐related behavior, early indicators of systemic toxicity [53, 54], (PMID: 34741005; PMCID: PMC8571423), were assessed monthly using the open‐field (OF) behavioral test. All behavioral evaluations were conducted by an operator blinded to experimental group allocation. Behavior was recorded and analyzed using the ANY‐maze Video Tracking System (Stoelting).

The OF test was performed as previously described [55]. Briefly, mice were placed in the center of a wooden arena (50 × 50 × 40 cm) with dark walls and allowed to explore freely for 10 min. The arena was virtually divided into three zones—periphery (within 10 cm of the walls), center, and corners—to assess anxiety levels and locomotor activity (Figure 18b). Total distance travelled, maximum speed, and time spent in each zone were automat^2^ically recorded. Time spent in the center zone was used an index of anxiety, since mice tend to spend more time in the periphery than in the center. The apparatus was thoroughly cleaned with 70% ethanol between sessions to eliminate olfactory cues.

#### 4.8.4 Tissue and Blood Collection

At three months post‐surgery, mice were euthanized under general anesthesia with inhaled isoflurane. To assess the biodegradation of the battery, the site of implant was recised and lifted and the absence of the battery was visually confirmed. Major organs, including the liver, spleen, kidney, heart, lungs, and brain, were collected, weighed, and observed to detect any potential macroscopic alterations. Blood was collected, allowed to clot at room temperature for 30 min, and then centrifuged at 1200 × *g* for 15 min to obtain serum for hepatic marker analysis.

#### 4.8.5 Hepatic Marker Analysis for Systemic Toxicity Evaluation

Serum alanine aminotransferase (ALT) activity was measured using a colorimetric assay kit (Elabscience Alanine Aminotransferase ‐ALT/GPT‐ Activity Assay Kit). Briefly, 5 µL serum per sample was analyzed, and absorbance was recorded at 510 nm using a Multiskan FC spectrophotometer (Thermo Scientific). ALT activity can be calculated by measuring the OD (Optical Density) values at 510 nm.

#### 4.8.6 Histological Analysis

Post fixed skin was immersed in 20% sucrose‐PBS for 2 days, and then 30% sucrose‐PBS for 3 days. The skin samples were frozen included in histo‐mount mounting medium using dry ice and stored at ‐80°C until use. Skin samples were sectioned (Leica CM1520 cryostat) at −27°C into 40 µm thick transversal slices and stored on slides at −20°C until use. The slides were air‐dried at room temperature for 4 h prior to hematoxylin and eosin histological staining that was conducted according to validated protocols [55]. Histological images were captured using an optical microscope (Nikon Eclipse Ni) with a 10X objective.

#### 4.8.7 Statistical Analysis

For in vivo and ex‐vivo studies, statistical analyses and graphs were performed using SPSS and GraphPad Prism software, respectively. Repeated‐measures ANOVA was applied; the type of implant was used as a factor and sex as a covariate. Statistical significance was defined as *p* < 0.05 (*p < 0.05; **p < 0.01; ***p < 0.001).

## Author Contributions

**Bincy Lathakumary Vijayan**: writing – original draft, investigation. **Hussien Hammoud**: investigation. **Marc Ramuz**: writing – review and editing. **Y. Tison**: writing – review and editing, methodology, investigation, resources. **E. Djenizian**: investigation. **Eleonora Vandini**: writing – original draft, investigation, methodology. **Lucas Teolis**: investigation. **Esma Ismailova**: writing – review and editing. **O. Jamai**: investigation. **Vedi Kuyil Azhagan Muniraj**: investigation, writing – review and editing. **Manuela Zavatti**: investigation. **S. Maria**: investigation. **T. Djenizian**: conceptualization, writing – review and editing, resources, supervision, project administration, validation, funding acquisition. **David Moreau**: writing – review and editing, methodology, resources. **H. Martinez**: writing – review and editing. **Daniela Giuliani**: writing – review and editing, supervision, resources.

## Funding

European Union Horizon Europe programme under grant agreement No 101046946 (RESORB) and by the Programme de Recherche PEPR Batteries France 2030 under grant agreement No ANR‐23‐PEBA‐0006 (SIMBA).

## Conflicts of Interest

The authors declare no conflicts of interest.

## Supporting information




**Supporting File 1**: advs76376‐sup‐0001‐SuppMat.docx.

**Supporting File 2**: advs76376‐sup‐0002‐VideoS1.mp4.

## Data Availability

The data that support the findings of this study are available from the corresponding author upon reasonable request.
