# Supplementary material for: Current Collector Engineering for New Efficient Bioresorbable Sodium‐Ion Batteries
Source: Adv Sci (Weinh). 2026 Jun 28:e76376. Online ahead of print. doi: 10.1002/advs.76376 (PMC13336757; doi:10.1002/advs.76376)
Supplement: Supplementary file 1 — Supporting File 1: advs76376‐sup‐0001‐SuppMat.docx. [file ADVS-9999-e76376-s001.docx]

Supporting information

Current Collector Engineering for New Efficient Bioresorbable Sodium-Ion Batteries

Bincy Lathakumary Vijayan^1†^, Eleonora Vandini^2†^, Vedi Kuyil Azhagan Muniraj^1^, Hussien Hammoud^1^, E. Djenizian^1^, Marc Ramuz^1^, Daniela Giuliani^2^, Manuela Zavatti^2^, Y. Tison^3^, H. Martinez^3,4,5^, Lucas Teolis^6^, Esma Ismailova^6^, David Moreau^6^, S. Maria^7^, T. Djenizian^1,8*^

^1^Mines Saint-Etienne, Center of Microelectronics in Provence, Department of Flexible Electronics, F-13541, Gardanne, France

^2^Department of Biomedical, Metabolic and Neural Sciences, University of Modena and Reggio Emilia, Via G. Campi 287, Modena 41125, Italy.

^3^ Université de Pau et des Pays de l’Adour, CNRS, IPREM, Pau, France.

^4^Centrale Casablanca, Research Center for Complex Systems and Interactions, Bouskoura Ville Verte, Maroc.

^5^Université Paris-Saclay CentraleSupélec, France

^6^Mines Saint-Etienne, Institut des Neurosciences de la Timone, Center of Microelectronics in Provence, Department of Bioelectronics, F-13541, Gardanne, France

^7^ Aix Marseille Univ, CNRS, ICR UMR 7273, Marseille, 13013, France

^8^Center of Physical-Chemical Methods of Research and Analysis, Al-Farabi Kazakh National University, Tole bi str., 96A. Almaty, Kazakhstan

*Corresponding Author E-mail Address [thierry.djenizian@emse.fr]

**TGA analysis of Quasi-solid-state electrolyte**


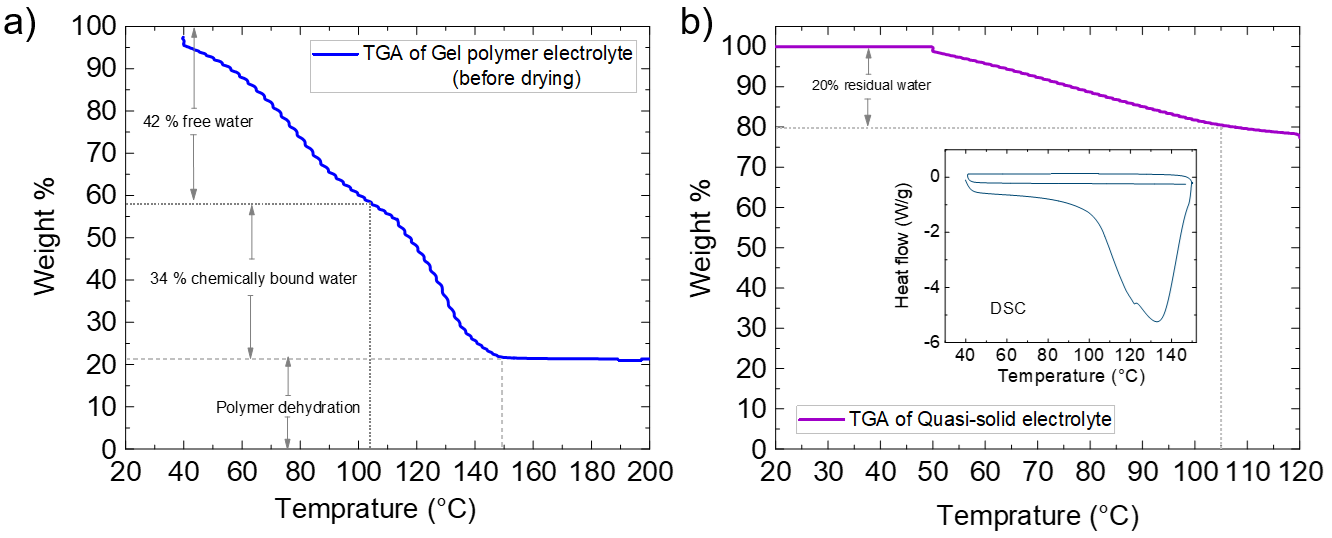

**Figure S1.** Thermal analysis of the gel and quasi-solid-state Na₂SO₄/Na-CMC electrolytes.

(a) TGA of the gel polymer electrolyte before drying, showing sequential mass loss. (b) TGA profile of the optimally dried to form QSSE. The inset shows the corresponding differential scanning calorimetry (DSC) curve of the QSSE.

**Electrical conductivity of electrodes with Mg and Mo-CC**

**
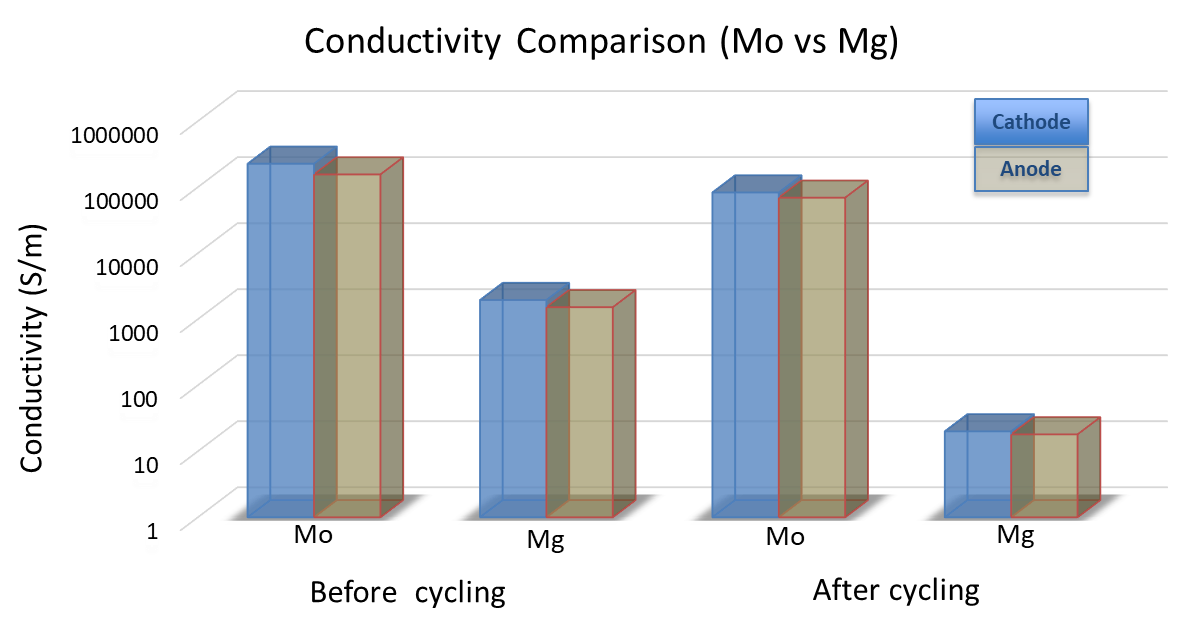
****Figure S2.** Comparison of electrical conductivity of cathode and anode current collectors (Mo and Mg) before and after cycling measured by four-probe conductivity method.

**Post cycling analysis of bioresorbable battery**


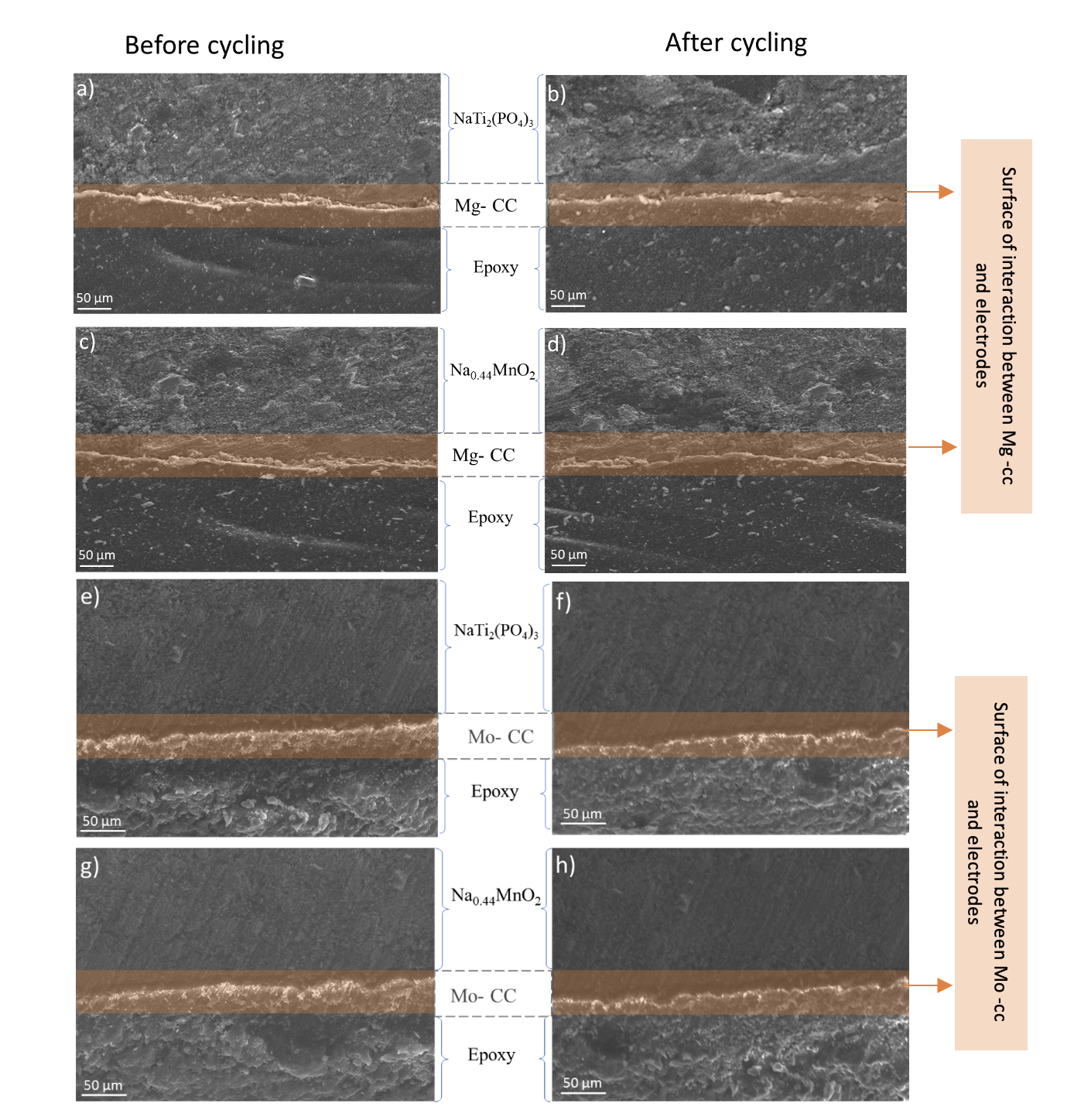


**Figure S3.** Cross-sectional SEM images of the interfaces between Mg and Mo current collectors (CC) and the corresponding electrodes before and after cycling. (a, b) Mg-CC / NaTi₂(PO₄)₃ (NTP-C) interface before and after cycling, respectively. (c, d) Mg-CC / Na₀.₄₄MnO₂ (NMO) interface before and after cycling. (e, f) Mo-CC/ NaTi₂(PO₄)₃ (NTP-C) interface before and after cycling. (g, h) Mo-CC / Na₀.₄₄MnO₂ (NMO) interface before and after cycling.
